# The Prenatal Environment in Twin Studies: A Review on Chorionicity

**DOI:** 10.1007/s10519-016-9782-6

**Published:** 2016-03-05

**Authors:** Kristine Marceau, Minni T. B. McMaster, Taylor F. Smith, Joost G. Daams, Catharina E. M. van Beijsterveldt, Dorret I. Boomsma, Valerie S. Knopik

**Affiliations:** Division of Behavioral Genetics, Department of Psychiatry, Rhode Island Hospital, Providence, RI USA; Department of Psychiatry and Human Behavior, Brown University, Providence, RI USA; Center for Alcohol and Addiction Studies, Brown University, Providence, RI USA; Department of Psychology and Child Development, California Polytechnic State University, San Luis Obispo, CA USA; EMGO Institute for Health and Care Research, VU University, Amsterdam, The Netherlands; Academic Medical Center, Medical Library, University of Amsterdam, Amsterdam, The Netherlands; Division of Behavioral Genetics, Coro West Suite 204, 1 Hoppin St, Providence, RI 02903 USA

**Keywords:** Chorionicity, Genetics, Heritability, Prenatal environment, Twins

## Abstract

A literature search was conducted to identify articles examining the association of chorionicity (e.g., whether twins share a single chorion and thus placenta or have separate chorions/placentas) and genetics, psychiatry/behavior, and neurological manifestations in humans twins and higher-order multiples. The main aim was to assess how frequently chorionicity has been examined in relation to heritability estimates, and to assess which phenotypes may be most sensitive to, or affected by, bias in heritability estimates because of chorionicity. Consistent with the theory that some chorionicity effects could lead to overestimation and others to underestimation of heritability, there were instances of each across the many phenotypes reviewed. However, firm conclusions should not be drawn since some of the outcomes were only examined in one or few studies and often sample sizes were small. While the evidence for bias due to chorionicity was mixed or null for many outcomes, results do, however, consistently suggest that heritability estimates are underestimated for measures of birth weight and early growth when chorionicity is not taken into account.

**Electronic supplementary material:**

The online version of this article (doi:10.1007/s10519-016-9782-6) contains supplementary material, which is available to authorized users.

## Introduction

Twin studies have long been used to estimate the unique contributions of genetic and environmental influences on variation in human traits. One assumption of the quantitative genetic theory underlying twin studies is the equal environments assumption, which states that the exposure to environmental events that create resemblance between co-twins for the trait under study is equal for monozygotic (MZ) and dizygotic (DZ) twin pairs (Loehlin and Nichols 1976; Scarr and Carter-Saltzman 1979). The prenatal environment is a specific and crucial environmental influence on many human traits (Barker 1990), and while twins and higher-order multiples share the womb, the prenatal environment may not be equal for both twins in a pair, or for other higher-order multiples. Thus, the prenatal environment cannot necessarily be considered as an environmental factor creating resemblance in children sharing the womb at the same time. How twins experience the prenatal environment depends, in part, on chorionicity, i.e., whether twins share a single chorion (monochorionic, MC) or have separate chorions (dichorionic, DC). Monozygotic (MZ) twins can be mono- or dichorionic, whereas dizygotic twins are dichorionic.

In this review, we first introduce the concepts of the chorion, amnion, and placenta. Next, we discuss how chorionicity may shape the prenatal environment of twins and higher-order multiples and aim to summarize the types of outcomes that have been linked to chorionicity. Finally we review and summarize studies which have examined the influence of chorionicity on twin-based heritability estimates in order to draw conclusions about whether chorionicity introduces bias and, if there is bias, whether this bias affects phenotypes in a consistent manner.

### Chorionicity

**Fig. 1:**
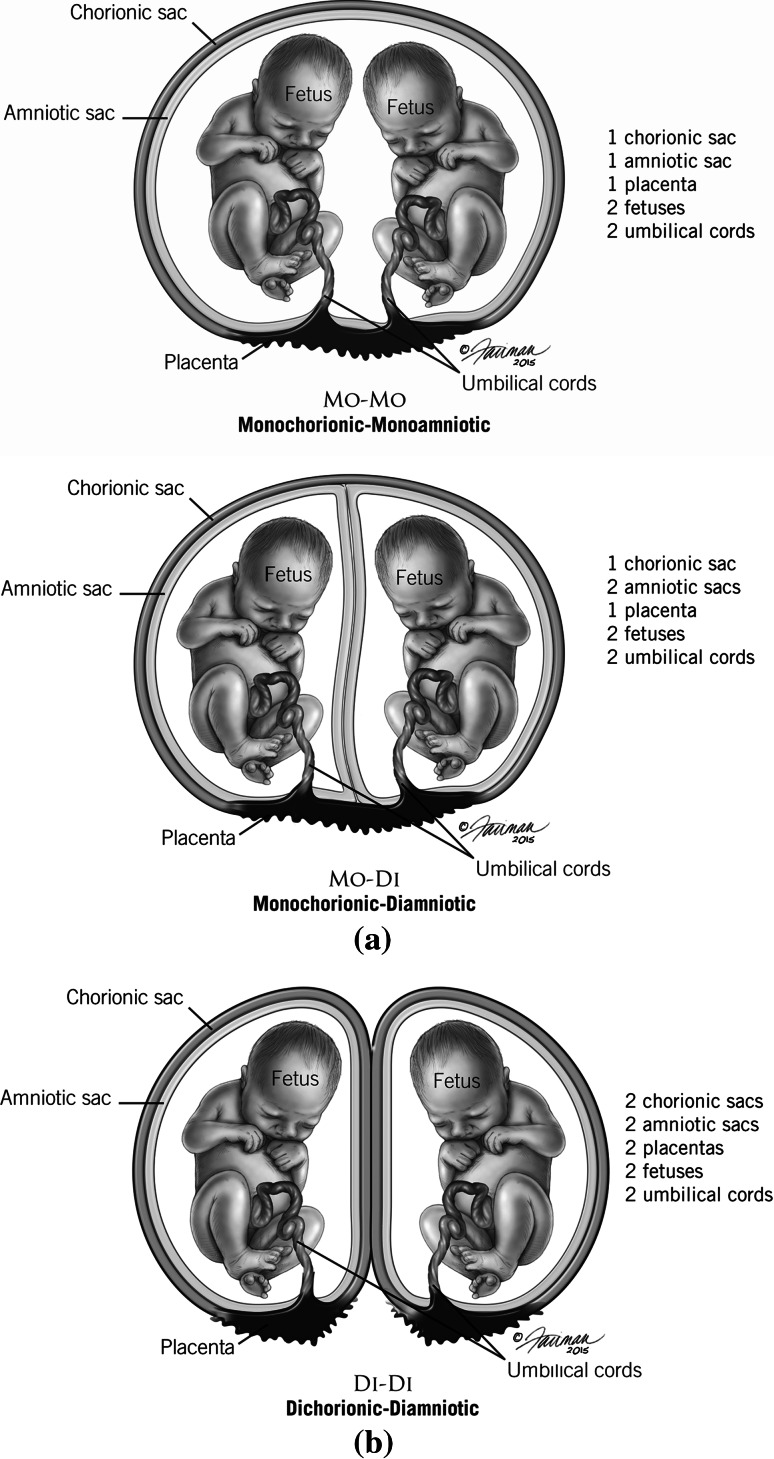
**a** Monochorionic-monoamniotic twins (MCMA, shown in the top image) have 1 chorion and 1 amnion. Monochorionic-diamniotic twins (MCDA, shown in the bottom image) have 1 chorion and 2 amnions. MC twins (whether MCMA or MCDA) share the same placenta. ©2015, Jennifer Fairman, CMI, FAMI. Published with permission. **b** Dichorionic-Diamniotic (DCDA) twins have two chorions and two amnions. Diamniotic twins can have the same or different placentas. © 2015, Jennifer Fairman, CMI, FAMI. Published with permission

**Fig. 2:**
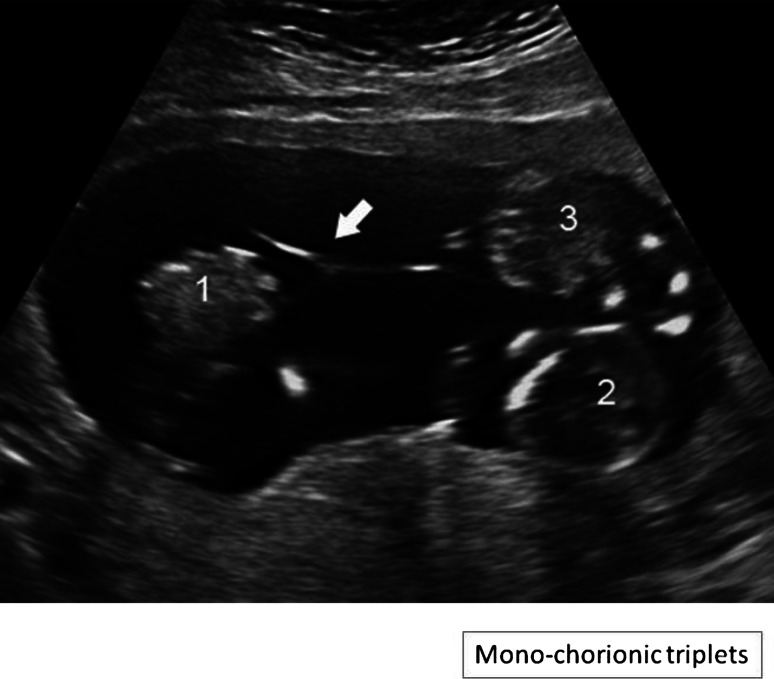
Ultrasound picture of a monochorionic, and therefore monozygotic trio at 12 weeks gestational age. The *arrow* indicates the meeting pointing point of three amniotic membranes. Numbers indicate the three fetuses. (Used with permission from Lamb et al. 2012)

**Fig. 3:**
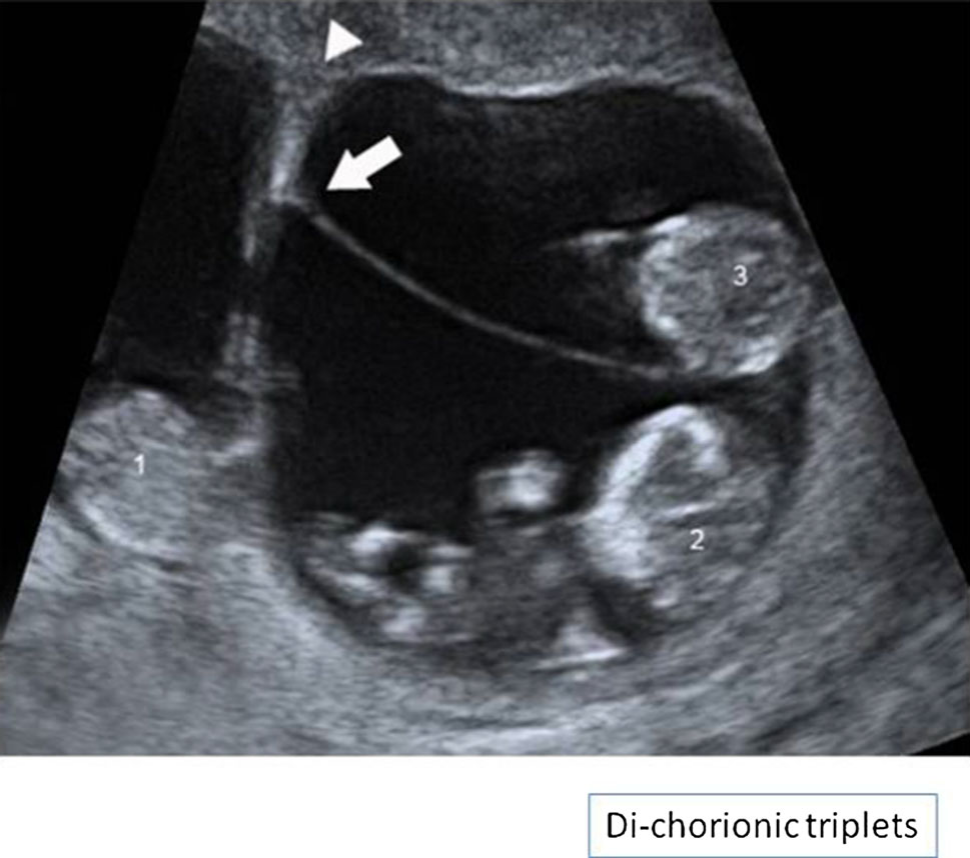
Ultrasound picture of a dichorionic, triamniotic trio at 13 weeks gestational age. The arrow indicates the amniotic membranes of fetuses 2 and 3, which are a monozygotic pair. At this time, it is unsure if Fetus 1 shares zygosity with fetuses 2 and 3. Numbers indicate the three fetuses. (Used with permission from Lamb et al. 2012)

**Fig. 4:**
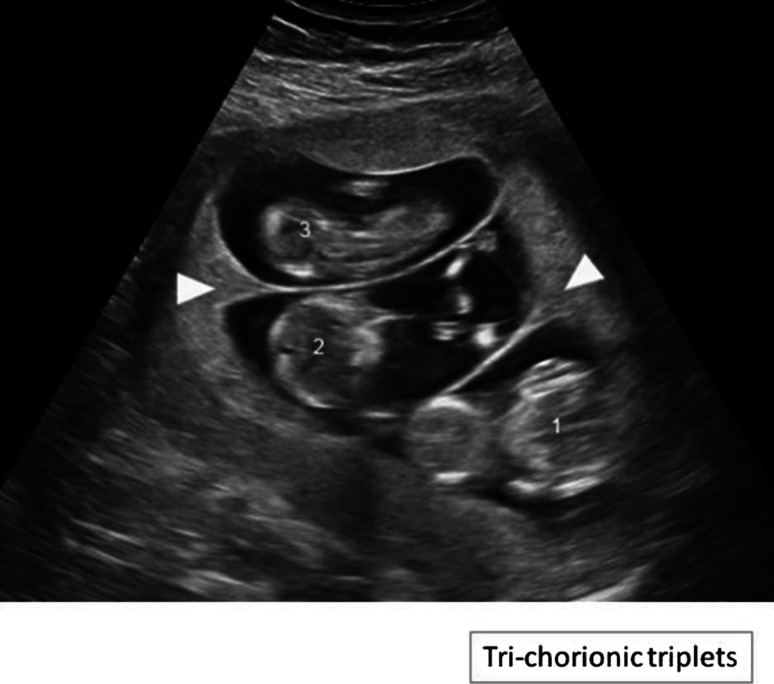
Ultrasound picture of a trichorionic trio at 12 weeks gestational age. These three fetuses do not share their placentas. This trio can be trizygotic, dizygotic (one identical duo), or monozygotic. Numbers indicate the three fetuses. (Used with permission from Lamb et al. 2012)

The chorion is the outer-most fetal membrane that contains the amnion/amniotic sac. The amnion is the thin inner-most fetal membrane that protects the embryo/fetus and contains amniotic fluid. The chorion connects the amnion, amniotic sac, and the fetus to the placenta and contributes to placental development. Thus, if twins share a chorion (e.g., are monochorionic or MC) they will share a single placenta, whereas twins with separate chorions (e.g., dichorionic or DC twins) develop individual placentas. DZ twins are dichorionic, since they form from two separately fertilized eggs, although very rare exceptions have been described in the literature (e.g., Souter et al. 2003). Figure 1a, b provides an illustration of the multiple ways co-twins can share the chorion and amnion. Figures 2, 3, 4 show ultrasound images of monochorionic (Fig. 2), dichorionic (Fig. 3), and trichorionic triplets (Fig. 4). Generally, it is thought that the timing of division of the blastocyst/embryo determines amnionicity and chorionicity (Hall 2003; De Paepe 2015), such that later cleavage (e.g., between 4 and 13 days) leads to MC twins and earlier cleavage (e.g., before 4 days) leads to DC twins. Later cleavage (e.g., 8–13 days) may lead to monoamniotic twins and earlier cleavage (e.g. before 8 days) to diamniotic twins. However, what determines whether and when a fertilized egg splits, and if the resulting MZ twins (or triplets or other higher order multiples) will develop separate chorions, are questions for which very little empirical data are available (Knopman et al. 2014; Herranz 2015).

### Prevalence

Epidemiological data indicate that the MZ twin prevalence is fairly consistent at around 4 per 1000 maternities worldwide (Tong et al. 1997). DZ twinning rates differ around the globe and over time (e.g., increasing with maternal age and as artificial reproductive techniques have become more widely available and used; Hoekstra et al. 2008). For example, among Caucasian populations (e.g., United States, Europe, Australia), total twinning rates were estimated at 15–16 per 1000 in 2003 (Hoekstra et al. 2008), whereas Asian countries had lower rates at about 9 per 1000 (Smits and Monden 2011; Hoekstra et al. 2008). African populations have higher twinning rates, of about 12–18 in sub-Saharan countries and over 18 per 1000 in central African countries (Smits and Monden 2011). Thus, in Caucasian and sub-Saharan African populations, MZ twins comprise ~26 % of all twins, whereas in Asian populations, MZ twins represent over half of all twins, and in central African populations MZ twins represent less than 5 % of all twins. Given that heritability estimates are specific to the population being studied, differences in the prevalence of MZ and DZ twins in different populations will likely affect the extent to which chorionicity might affect heritability estimates in these populations.

Of all MZ twin pairs, about two-thirds (70–74 %) are monochorionic (MZ-MC) and one-third (35–30 %) are dichorionic (MZ-DC) (Hall 2003). However, 1–2 % of MZ twin pairs are monoamniotic (Hall 2003) although this percentage varies by sample. Given the low prevalence of monoamniotic twins this review focuses on the potential effects of chorionicity rather than amnionicity. For Caucasian populations (where most twin research has been done) about 17 % of all twin pairs are MZ-MC, ~9 % are MZ-DC, and ~74 % are DZ-DC. However, the proportion of MZ-MC, MZ-DC, and DZ-DC twins in any given study varies widely and is not always reported (Petterson et al. 1998).

### Determination

A large body of literature has examined appropriate ways to determine chorionicity. Prospectively, chorionicity is best determined via ultrasound. Determining chorionicity is highly accurate (96 %) by ultrasound in the first trimester, though still accurate (80 %) in the second (e.g., see Audibert and Gagnon 2011 for review; Machin 2004). Placental pathology examination also provides a direct assessment of chorionicity shortly after birth (De Paepe 2015). Retrospective self-report determination of chorion type, for example by asking twin participants “how many placentas” there were at birth, has been suggested to be unreliable: 60 % accurate for MZ and 37 % accurate for DZ twins (Derom et al. 2003). Some studies have also tried to use dermatoglyphics to retrospectively determine chorionicity (e.g., Davis et al. 1995; Reed et al. 1991, 1978, 2002; Melnick and Myrianthopoulos 1979; Steinman 2001). Placental pathology examination and ultrasound appear to be the most reliable methods of determining chorionicity; thus, for the remainder of this paper we focus on studies which employed one of these two methods.

### Placental function

The MC placenta functions like a single placenta, although a single placenta was not designed to support the growth of two fetuses. Therefore, MC placentation has a profoundly different biology than DC placentation. The greatest danger associated with MC placentation is related to the structure of blood vessels. One twin usually has better placement and therefore receives more of the nutrients. Inter-fetal vascular connections also form vascular anastomoses (i.e., the joining of two blood vessels) and connect the circulation of one twin to the circulation of the other, so in some pregnancies, there is direct blood sharing of MC twins. These inter-fetal vascular connections rarely form in DC twin pairs (Machin and Bamforth 1996; Phillips 1993).

Unequal placental sharing is a major cause of fetal growth discordance in MZ twins (Chang 2008; Cleary-Goldman and D’Alton 2008; Nikkels et al. 2008). For example, specific reductions in five amino acids have been shown to explain discordant growth in MZ twins, suggesting that the inter-twin distribution of blood and nutrients accounts for within-pair differences in birth weight, as opposed to more general placental dysfunction (Bajoria et al. 2001). Extreme discordant growth due to unequal placental sharing can result in twin-to-twin transfusion (TTTS) syndrome, a severe pregnancy complication unique to MC twin pairs where there is also direct blood sharing (occurring in 5–30 % of MC twin pairs; Haverkamp et al. 2001; Phillips 1993). The imbalanced blood flow and twin-to-twin transfusion has been reported to influence MZ twin resemblance for birth weight (see Foley et al. 2000 for review, and supplemental Table 1). These findings result in a difference in MC and DC twins for some birth outcomes including birth weight discordance, as MC twins are more likely to have higher birth weight discordance than DC twins who do not share a placenta.

**Table 1:** Mechanisms of potential bias in heritability estimates due to chorionicity

Mechanism of chorionicity effects	MC and DC twin similarity	Bias in heritability estimate	Rationale
Vascular differences: placental sharing inequalities	MC twins less similar than DC twins	Underestimated	MZ twins would have lower correlation, closer to DZ twins (reducing contrast)
Similar placental function: diffusion, osmosis, endocrine	MC twins more similar than DC twins	Overestimated	MZ twins would have higher correlations than DZ twins, chorionicity effect would be included in heritability estimate
Mis-classification of MZ and DZ twins	MC twins that are less similar may be called DZ instead of MZ twins	Most likely underestimated	Including MZ twins in DZ group would mean more genetic similarity in DZ group, reducing contrast in twin correlations
MC twins have poorer outcomes than DC twins	MC twins less or more similar to DZ twins	Underestimated or Overestimated	MC twinning is indicative of a prenatal environmental risk factor(s). If the MC twinning environmental factor(s) is shared, MZ twins would have a higher correlation than DZ twins; if the MC twinning factor was unshared, the MZ twins would have a lower correlation, closer to DZ twins.

The placenta also functions as a barrier, allowing small molecules (e.g., gases, nutrients, waste material, antibodies) to pass between mothers and children through passive transport (Page 1993; Schneider 1991). Other small molecules that may have an effect of fetal development (e.g., some maternal hormones like cortisol; bacteria; teratogens such as illicit drugs) can also be diffused through the placenta (van der Aa et al. 1998; Page 1993). Thus, the composition of the placenta and efficiency of transport between mother and child can affect fetal development. The placenta also functions as an endocrine organ (Melmed et al. 2012), synthesizing a large array of hormones (e.g., sex steroids and protein hormones) and cytokines that play a key role in fetal development (and maternal endocrine function). There are individual differences in hormone production, and sharing a placenta may lead to similarities in MC twins that are related to the levels and changes in placental hormone production relative to DC twins. Sharing a placenta in this case may lead to more similar in utero environments for MC twins relative to DC twins. However, endocrine function is, to some extent, linked to the vascular system, and the amount of pathogen, infection, nutrient, and gas and waste diffusion may also be linked to the proportion of the placenta dedicated to each child (Melmed et al. 2012). The potential impact of diffusion and endocrine function on similarity and differences of MC versus DC twins has not, to our knowledge, been investigated and is potentially an important area for future research. Thus, while some placental mechanisms (diffusion and endocrine function) may lead to more similar whereas others (unequal sharing of the vascular system) may lead to more different in utero environments, these mechanisms are linked and so the reality is less clear-cut.

### Chorionicity and heritability

Because of the placental mechanisms leading to similarities and differences of the in utero environments for twins of different types, chorionicity may bias the heritability estimates found in twin studies (see Table 1). The potential challenge that chorionicity plays in the validity of twin studies is not a new concept (Price 1950), and has been highlighted in a number of studies (Derom et al. 2001; Foley et al. 2000; Munsinger 1977; O’Brien and Hay 1987; Phelps et al. 1997; Prescott et al. 1999; Price 1950). The prenatal environment could be more similar for MC twins relative to DC twins because of the shared chorion, or less similar because of the vascular and placental sharing inequalities often observed in MC but not DC pregnancies. Vascular differences found in MC twins often lead to differences in intrauterine growth of the twins, and thus MC twins can appear quite dissimilar especially early in life. If zygosity is only determined via questionnaire, MC twins may be misclassified as DZ twins, which would bias results of twin studies (Machin 2001, 2009). Even with correct classification, if MC twins are more dissimilar because of unequal placental sharing, then heritability estimates may be underestimated because MZ twins would have a lower correlation, closer to that of DZ twin pairs (Price 1950). That is, the subset of MZ-DC twins may be more similar to DZ-DC and less similar to MZ-MC twins in their sibling correlations. This would, in turn, affect the intra-class correlations for MZ and DZ pairs (e.g., reduce the contrast) and downwardly bias the estimates of heritability. Further, MC twins often have poorer outcomes than DC twins (see review below and Supplemental Table). This may lead to mean-level or variance differences in the outcomes between MC and DC twins due to a possible violation of the equal environments assumption, which could also bias heritability estimates. For example, if in a pair of MC twins, one of the twins is at increased risk for a particular outcome (e.g., through limited blood supply because of TTTS), then the prenatal environment is not ‘shared’ although the MC status is considered ‘shared’.

However, if sharing a placenta makes twins more similar because of similar intrauterine environments (e.g., passive transport), then the potential bias could indeed operate in the opposite direction, leading to overestimation of genetic influences (Phillips 1993). For example, MC pairs may be more likely to experience the same environmental exposures and pathogens, including infections and substance use exposure (Prescott et al. 1999). The crux of understanding how chorionicity may influence heritability estimates lies in understanding whether the prenatal environment is more or less similar for MC twins, and for which outcomes chorionicity matters for twin similarity.

This ‘chorionicity debate’ led to the proposal for chorion-control studies, where MZ-MC twins are compared with MZ-DC twins on a specific trait, or multiple traits, and a call for including chorionicity in classical twin studies (Phelps et al. 1997). However, methodological challenges have made the examination of the potential role of chorionicity difficult and largely theoretical; as noted above, a reliable assessment of chorionicity ideally requires placental pathology examination or prenatal ultrasound. As there is an increasing interest in simultaneously examining prenatal and genetic influences as exemplified in this special issue of *Behavior Genetics*, it is important to revisit the question of whether chorionicity may influence outcome variables assessed in twin studies and whether such influence could bias heritability estimates from studies that include predominantly twins.

## Method

### Medical library database search

The purpose of the literature search was to identify articles examining associations of chorionicity and genetics, psychiatry/behavior, and neurological manifestations in humans (twins/multiples). We searched PubMed (yielding 2111 articles after deleting duplicates), Embase, 1947 to present, OvidSP (yielding 1455 articles after deleting duplicates), and PsycINFO 1806 to Present (yielding 138 articles after deleting duplicates). The entire search strategy, including all search terms for each database, is included in Appendix. A variety of search terms were used (both text words [tw] and the PubMed search also included Medical Subject Heading terms [MeSH]), including but not limited to variants of multiple birth (e.g., multiple birth, twin), chorionicity (e.g., chorion, monochori*, dichori*, placentation), genetics (e.g., genetic*, epigenetic*, gene, genes, genotype), intelligence (e.g., intelligence, IQ), psychiatry/behavior (e.g., psychology, psychiatry*, mental, psychology*, behavior, neuropsych), neurological manifestations (e.g., neuromorbidity, neurologic*), and concordance/discordance (e.g., twin, discordan*, concordan*). In Embase, twin concordance and discordance was searched in combination with the outcome separately because of poor representation of chorionicity in the bibliographic records. Animal studies were excluded in all searches. We did not filter by language or date of publication. After duplicates from the multiple searches were excluded, there were a total of 2920 unique articles.

### Selecting relevant articles

Each of the abstracts of the 2920 articles were read and judged for relevance to chorionicity and genetics/behavior/psychiatry (e.g., identifying sources which examine the association of chorionicity with behavioral outcomes). Full texts were also searched for “chor” to aid with determining whether articles were relevant. Case studies and non-empirical articles were excluded from the final selections. We also excluded studies that used retrospective report of chorionicity as well as other alternative proxies for chorionicity (e.g., birth weight discordance, handedness, mirroring). At the end of this culling, 307 articles were identified as potentially relevant.

These 307 articles were further classified into background/review articles (*n* = 68), studies that compare the prevalence of various outcomes stratified by chorionicity (reviewed below and in the Supplementary Table, *n* = 134), studies that examined chorionicity effects in the context of behavioral genetic designs (*n* = 38), epigenetic studies (*n* = 5), and irrelevant studies (e.g., not examining chorionicity directly, or conference abstracts which may be preliminary and not peer reviewed, vetted findings, *n* = 62). This sorting was done by reading the abstracts and articles to the depth required to make a decision. Of primary interest for the current review were the studies that examined chorionicity effects in the context of behavioral genetic designs. These studies were reviewed in detail in order to conclude whether chorionicity may bias results of heritability estimates for the diverse outcomes studied. We did not restrict our search based on outcomes during this phase.

## Results

### Chorionicity and prevalence of birth outcomes and human traits

A very large body of literature has examined whether there are prevalence differences in various birth, perinatal, and other outcomes based on chorionicity (see Supplementary Table for a summary of the 134 articles reviewed). The best-characterized outcomes influenced by chorionicity include immediate pregnancy and birth outcomes rather than longer term growth and psychiatric outcomes. We highlight the most consistent findings here (see Supplementary Table for details and exceptions). Most studies found that MC pregnancy infers higher risk of mortality than DC pregnancies (see Supplementary Table), but effects are not always consistent (e.g., Baghdadi et al. 2003; Lenis-Cordoba et al. 2013). Fetal growth has also been robustly linked with chorionicity. For example, birth weight discordance occurs more frequently in MC twins than DC twins (although this effect is not found in every study). Further, MC twins generally have lower birth weight (especially the smaller twin), lower birth weight after adjusting for gestational age (Ananth et al. 1998; Shrim et al. 2010), and shorter crown-rump length. Intrauterine growth restriction is more prevalent in MC twins than DC twins. However, fetal growth velocity has not been shown to differ for MC versus DC twins (Smith et al. 2001; Taylor et al. 1998). A host of obstetric and perinatal complications have also been examined extensively in relation to chorionicity. Most studies have found that DC twins are born at older gestational ages than MC twins, and experience fewer morbidities (e.g., patent ductus arteriosus, sepsis, vision and auditory loss, congenital malformations, anemia, intracranial lesions). In general, MC pregnancies are riskier than DC pregnancies.

In contrast to pregnancy and birth outcomes, associations of chorionicity and cognitive, psychiatric, and behavioral outcomes are not as frequently studied or as consistent. The limited literature hints that MC twins have worse cerebral white matter outcomes than DC twins. For example, MC twins have higher cerebral white matter lesions (Adegbite et al. 2005) and a higher incidence of antenatal necrosis of cerebral white matter (Bejar et al. 1990) than DC twins. However, another study showed no differences in clinical neurologic indicators of perinatal asphyxia (van Steenis et al. 2014). In terms of cognitive performance, results are mixed. One study suggested that MC twins have higher rates of pathological nonverbal performance and learning disabilities (Einaudi et al. 2008), whereas other studies showed no difference in mental development indexes (e.g., on the Bayley; Welch et al. 1978; Steingass et al. 2013). Studies examining cerebral palsy are inconsistent, with some suggesting that MC twins are at a higher risk (Burguet et al. 1995, 1999), but others finding no difference in prevalence of cerebral palsy in MC versus DC twins (Steingass et al. 2013; Hack et al. 2009), or that the association was attenuated when controlling on other perinatal factors (Livinec et al. 2005).

### Chorionicity and behavioral genetic designs

We identified 38 articles that examined chorionicity within a behavioral genetic design. Of these, one was excluded because no full text was available in English. An additional seven were excluded because chorionicity was not determined via placental pathology or ultrasound. We organized the resulting 30 studies into the following outcome-based categories (although some studies have multiple outcomes across multiple categories): birth weight and early growth, screening/vaccination, handedness, anthropomorphic measures, cognitive/brain measures, and behavioral measures. Reviewed studies are presented in Table 2.

**Table 2:** Reviewed studies examining chorionicity with a behavioral genetic design

Reference	N (twin pairs)^a^	Location	Placentation determination	Age	Comparison	Analysis	Outcome	**Effect**	**Other notes**
Birth weight (BWT), early growth									
Buzzard et al. (1983)	52 MZMC 40 MZDC 72 DZDC	Canada	Placental pathology	20–41 years	MC versus DC and MZ versus DZ	Intra-pair differences	BWT	MC = DC; MZ = DZ	Trends for intra-pair differences in chorionicity and chorionicity by sex interaction
Vlietinck et al. (1989)	762 MZ 1093 DZ	Belgium	Examination of placenta	Birth	MZMC versus MZDC versus DZ	Extended ACE model with chorionicity included	BWT	*r*MC < *r*DC (MC also less variable than DC)	EFPTS
Gielen et al. (2008)	4232 twin pairs	Belgium	Examination of placenta	Birth	MZ versus DZ, chorionicity a covariate	ACE models, chorionicity a covariate	BWT	Removing chorionicity resulted in a decrease of genetic variance and heritability	EFPTS
Touwslager et al. (2010)	522 individuals	Belgium	Examination of placenta	0–2 years	MZMC versus MZDC versus DZ	Intra-pair growth correlations	Growth during infancy	*r*MZMC = *r*MZDC at 0-1, 0-6, or 6-12 months; *r*MZMC > *r*MZDC at 12-24 months;	EFPTS; MZMC grew more slowly than MZDC
Misc. screening/vaccination									
Wojdemann et al. (2006)	31 MC 150 DC	Denmark	Ultrasound	Birth	MC versus DC	ICCs	Nuchal translucency	*r*MC = *r*DC	
Gupta et al. (2008)	117 DC 16 MC	India	Examination of placenta	Newborns	MC versus DC and MZ versus DZ	Intra-pair agreement	BCG vaccine reaction	*r*MC = *r*DC; *r*MZ = *r*DZ	
Handedness									
Carlier et al. (1996)	20 MZMC 24 MZDC 24 DZ	France	Examination of placenta	8–12 years	MZMC versus MZDC versus DZ	Comparison of difference between groups on the average absolute twin difference; if chorion effect not significant, all MZs pooled for MZ/DZ comparison	Manual performance, direction, laterality (handedness)	No chorionicity effects	*r*MZ = *r*DZ in classic twin design (chorionicity not controlled)
Melnick and Myrianthopoulos (1979)	117 MZMC 56 MZDC	USA	Sex, nine blood groups, and gross and microscopic examination of placenta	Birth through 7 years	MZMC versus MZDC	Comparing concordance	Finger ridge count and right-left asymmetry of ridge count and congenital anomaly (e.g., differentiation of body parts, tissue differentiation or dysplasias)	*r*MZDC < *r*MZMC for total ridge count. No chorionicity effects for congenital anomalies, other assessments of dermatoglyphics	NCPP twin population
Anthropomorphic measures									
Corey et al. (1976)	30 MZMC 22 MZDC	USA	Examination of fetal membranes	Newborns	MZMC versus MZDC	General linear model for twin data, applied to MC and DC twins	Cholesterol from cord blood	Chorion type had a sig effect on within-pair not among-pair variation. *r*MC > *r*DC	Female MZ > male MZ for cord blood cholesterol
Hur and Shin (2008)	81 MCMZ 47 DCMZ 457 DZ	S. Korea	Examination of placenta	2–9 years (mean = 4 years)	MZMC versus MZDC versus DZ	ACE models including chorion effects	Height, weight, BMI	A + C sig. chorion effects for height sig but only 4 %, not sig for weight/BMI	
Blekher et al. (1998)	17 MZMC 16 MZDC	USA	Examination of placentas	9–18 years (mean 13.2 years)	MZMC versus DC	ICCs	Saccadic eye movements: accuracy, slope, velocity at 15 degree saccade	*r*MZMC > *r*MZDC for reaction times; not phasic component of saccadic command.	Follows up a traditional MZ/DZ study showing sig. genetic influence
Souren et al. (2007)	240 MZ 138 DZ	Belgium	Examination of placenta	Adults	MZMC versus MZDC	ICCs	Body mass, BMI, fat mass, waist-to-hip ratio, sum of four skinfold thickness, lean body mass, fasting glucose, fasting insulin, insulin resistance and beta cell function, and insulin-like growth factor binding protein-1 levels, total, LDL, HDL, total:HDL ratio for cholesterol, triacylglycerol, NEFA and leptin levels	*r*MZMC = *r*MZDC	EFPTS; regular twin study run subsequently
van den Borst et al. (2012)	165 MZ 71 DZ 102 single twins	Belgium	Examination of placenta	Adults	MZMC versus MZDC	ICCs	Lung measures: forced expiratory volume in 1 s and forced vital capacity	*r*MZMC = *r*MZDC	EFPTS; regular twin study run subsequently
Fagard et al. (2003)	125 DZ 97 MZDC 128 MZMC	Belgium	Examination of placenta	18–34 years	MZMC versus MZDC versus DZ	Intra-pair correlations; ACE models fit to MZ/DZ; MZDC/DZ; MZMC/DZ	Conventional and ambulatory blood pressure	*r*MZMC = *r*MZDC Heritability estimates similar when MZ/DZ or MZMC/MZDC	EFPTS
Loos et al. (2001a)	280 MZMC 212 MZDC 311 DZ	Belgium	Examination of placenta	18–34 years	MZMC versus MZDC versus DZ	Intra-pair concordance	Abdominal obesity	Contribution of zygosity and chorionicity low (< 1.7 %) for adult weight. *r*DC > *r*MC for BWT only	EFPTS
Loos et al. (2001b)	67 MC 56 DC	Belgium	Examination of placenta	*M* = 25 years	MZMC versus MZDC versus DZ	ICCs	Fasting fibrinogen	*r*MZMC < *r*MZDC Suggested chorionicity effect would change heritability	Article type = “Correspondence” about EFPTS *r*MZ > *r*DZ
Cognitive and brain measures									
Mukherjee et al. (2009)	22 MZMC 17 MZDC 49 DZDC	USA	Placental pathology, or ultrasound if pathology not performed or unavailable	Prenatal and birth	MZMC versus MZDC versus DZ	Comparing concordance	Head circumference and weight at 22 weeks, 32 weeks, birth, and intracranial volume	*r*MZMC = *r*MZDC head circumference and weight; Sig. group difference on ICV (DZ > MZMC and MZDC)	
Welch et al. (1978)	20 MZMC 12 MZDC	USA	Gross and microscopic placental examination	18 months	MZMC versus MZDC	Differences in within-pair mean squares	BWT, Bayley mental development scores	*r*DC > *r*MC for BWT; not significant (in opposite direction) for Bayley	Positive correlation of BWT and Bayley
Melnick et al. (1978)	86 MZ 173 DZ	USA	Sex, nine blood groups, and gross and microscopic examination of placenta	7 years	MZMC versus MZDC and MZDC versus DZ	Heritability estimates	IQ	heterogeneity between MZMC/DZ total variances, but not MZDC/DZ. Estimated genetic variance comparing MZMC/DZ not sig diff from 0, but MZDC/DZ was.	NCPP twin population, also split by race
Sokol et al. (1995)	23 MZMC 21 MZDC	USA	Gross and microscopic examination of placentas	~6 years	MZMC versus MZDC	Intra-pair differences	McCarthy Scales of Children’ s Abilities and Personality Inventory for Children	*r*MZMC > *r*MZDC for 3/4 Personality Inventory for Children factor scales, 8/12 clinical scales, and 2/4 validity/screening scales. No differences on 6 McCarthy scales.	
Antoniou et al. (2010)	663 twin pairs	Belgium	Examination of placenta	7–15 years, *M* = 10.4 years	MZ versus DZ on placentation	Bivariate ACE models	BWT-IQ and cord knots-IQ associations	No chorionicity effect	
Spitz et al. (1996)	20 MZMC 24 MZDC 24 DZ	France	Ultrasound and macroscopic examination at delivery	8–12 years	MZMC versus MZDC	Intra-class correlations	Anthropometric measures at birth and assessment; Cognitive battery (Vocabulary, block design from WISC-R, K-ABC, perception and mental rotation	*r*MC < *r*DC for BWT, child weight, height, BMI. *r*MC > *r*DC block design. No chorion effect for vocabulary, K-ABC, perception, or mental rotation	
Jacobs et al. (2001)	161 MZMC 82 MZDC 188 DZ	Belgium	Examination of placenta	9–11 years	MZMC versus MZDC versus DZ	ACE models including chorion effects	WISC-R	*r*MZMC > *r*MZDC on arithmetic (chorionicity = 14 % of total variance) and vocabulary (chorionicity = 10 %)	EFPTS; High heritability for almost all subscales and IQ scores.
Gutknecht et al. (1999)	20 MZMC 23 MZDC	France	Examination of placenta	10–16 years	MZMC versus MZDC	Comparison of average within-pair difference between groups	Weight, height, BMI; WISC-III; figurative reasoning; schools’ standardized exams; Personality and behavioral measures	*r*MC > *r*DC weight, BMI; 1/6 WISC-III measures; figurative reasoning, 1/5 personality/behavioral variables (most null)	Lack of power; but found consistent direction of effects in even null findings
Rose et al. (1981)	17 MZMC 15 MZDC 28 DZ	Canada	Examination of placenta	Adults	MZMC versus MZDC versus DZ	Mean differences, ICCs, heritability estimates (from several contrasts)	WAIS vocabulary and block designs	*r*MZMC > *r*MZDC (~ 2x) for block design only; vocabulary: *r*MZMC = *r*MZDC > *r*DZ	
Melnick et al. (1980)	94 MZ 187 DZ	USA	Gross and microscopic examination of placenta	4 months and 1 year	MZ versus DZ, checked chorion effects	Within-pair mean square estimates of genetic variance	Anterior fontanelle development	No chorionicity effects at 4 months or 1 year	NCPP twin population, also split by race
Other behavioral measures									
Chen et al. (1990)	44 MZ 18 DZ	China	Examination of placenta	6 months	MZ versus DZ and MZMC versus MZDC	Intra-pair correlations	Temperament	*r*MZMC = *r*MZDC	Found genetic influences
Hur (2007)	56 MZMC 34 DCMZ 316 DZ	S. Korea	Examination of placenta	2–9 years (*M* = 4 years)	MZMC versus MZDC versus DZ	ACE models including chorion effects	Prosocial behaviors	No chorionicity or shared environment effects.	Twin study estimates good
Riese et al. (1999)	48 MZMC 29 MZDC	USA	Examination of placenta	Neonates before released from hospital	MZMC versus MZDC	ICCs	Temperament	*r*MZMC = *r*MZDC	
Wichers et al. (2002)	202 MZMC 125 MZDC 425 DZ	Belgium	Examination of placenta	6–17 years	MZMC versus MZDC versus DZ	ICCs, ACE model	CBCL total problems score	*r*MZDC = *r*MZMC > *r*DZ. Chorionicity effect was 0 in the full ACE model	EFPTS

Described effects are not necessarily comprehensive. Findings were interpreted by comparing MC and DC similarity (e.g., ICCs)—with MC and DC similarity denoted by *r* (e.g., the within-twin pair correlation)

^a^ *N* number of twin pairs unless otherwise noted, *MZMC* monozygotic monochorionic twins, *MZDC* monozygotic dichorionic twins, *DZDC* Dizygotic dichorionic twins, *BWT* birth weight, *MC* monochorionic, *DC* dichorionic, *MZ* monozygotic, *DZ* dizygotic, *EFPTS* East Flanders Prospective Twin Survey, *ICC* intra-class correlation, *BCG* Bacille Calmette-Guerin vaccine, *NCPP* NINCDS Collaborative Perinatal Project, *BMI* body mass index, *LDL* low density lipoprotein, *HDL* high density lipoprotein, *NEFA* non-esterified fatty acids, *ICV* intracranial volume, *BWT* birth weight, *WISC* Wechsler Intelligence Scale for Children, *K-ABC* Kaufmann Assessment Battery for Children, *WAIS* Wechsler Adult Intelligence Scale, *CBCL* Child Behavior Checklist

Eight studies examined chorionicity effects on intra-pair associations/differences and/or included chorionicity in classical twin models decomposing the variance in a phenotype into additive genetic (A), common environmental (C), and non-shared environmental (E) influences (e.g., ACE models) in regard to birth weight and early growth patterns. Across these studies, generally it was found that MC twins grew more slowly, were less variable, and less correlated for birth weight than DC twins, and that including chorionicity yielded attenuated, more precise heritability estimates (Buzzard et al. 1983; Vlietinck et al. 1989; Gielen et al. 2008; Touwslager et al. 2010; Welch et al. 1978; Mukherjee et al. 2009; Spitz et al. 1996; Loos et al. 2001a). Although effects were not always significant (e.g., trend-level; Buzzard et al. 1983), the evidence does point to biased heritability estimates in studies of birth weight; where, without accounting for chorionicity, heritability is underestimated.

One study examined screening for trisomy 21 and one examined responses to vaccination (Wojdemann et al. 2006; Gupta et al. 2008). Neither study found evidence of a chorionicity effect on twin similarity. Two studies examined handedness (Carlier et al. 1996; Melnick and Myrianthopoulos 1979). Neither found any effects of chorionicity on twin similarity.

Eleven studies measured various anthropometric measures. Chorionicity effects varied with outcome and over time. For example, MZ-DC twins were more discordant for cholesterol levels from cord blood than MZ-MC twins (Corey et al. 1976). There were significant chorionicity effects when modeled explicitly for height at age 4 years, explaining a small percentage of variance (4 %), but not for weight (Hur and Shin 2008). One study suggested that MZ-MC twins were more discordant than MZ-DC twins for height at 8–12 years (Spitz et al. 1996), however another found that there were no differences in the concordance of MZ-MC and MZ-DC twins for height in at 10–16 years (Gutknecht et al. 1999). MZ-MC twins were more discordant than MZ-DC twins for weight and BMI throughout childhood and adolescence (Gutknecht et al. 1999; Spitz et al. 1996; Mukherjee et al. 2009). There was also some evidence that MZ-MC twins were more similar than MZ-DC twins for saccadic eye movements in adolescence (Blekher et al. 1998). In adults, there were no differences in the twin similarity of various obesity-related measures (or very small effects; Loos et al. 2001a), lung measures, or conventional and ambulatory blood pressure (Loos et al. 2001a; van den Borst et al. 2012; Souren et al. 2007; Fagard et al. 2003). The only significant chorionicity effect on twin similarity found in adults was for fasting fibrinogen: MZ-DC twins were more similar than MZ-MC twins (Loos et al. 2001b). In sum, chorionicity appears to maintain an effect on twin similarity for a variety of anthropometric measures even after birth, but these effects seem to dissipate in later adolescence and adulthood. However the directions of effects varied for each measure. Based on the limited evidence provided here, heritability estimates may be overestimated for cord blood cholesterol, saccadic eye movements, and height at age 4 years. However, heritability estimates may be underestimated for height at 8–12 years, weight and BMI in childhood and adolescence, and fasting fibrinogen in adults.

Eight studies examined cognitive and brain-based measures, and findings were generally mixed. Studies very early in life (e.g., from in utero to 1 year) found no significant effects of chorionicity on twin similarity for head circumference, intracranial volume (Mukherjee et al. 2009), or anterior fontanelle development (Melnick et al. 1980). In toddlerhood, there were no chorionicity effects on twin similarity for the Bayley Mental Development scores (Welch et al. 1978). In childhood, there was evidence of two populations of MZ twins with regard to variation in IQ, as MZ-MC twins differed from DZ twins but MZ-DC twins did not (Melnick et al. 1978), suggesting considerable influence of the prenatal environment on IQ. However, another study showed that there were no differences in twin similarity based on chorionicity for the McCarthy Scales of Children’s Abilities (Sokol et al. 1995). Also in childhood, one study found that MC twins were more similar for arithmetic and vocabulary (with chorionicity explaining 14 and 10 % of the total variance respectively; Jacobs et al. 2001), whereas another found no effect of chorionicity on twin similarity for vocabulary (Spitz et al. 1996). MZ-MC twins were more similar than MZ-DC twins for measures of personality in one study (Sokol et al. 1995), whereas another study found null findings for measures of personality (Gutknecht et al. 1999) in childhood. Some studies found relatively few significant effects of chorionicity on twin similarity (relative to the number of tests examined, e.g., Gutknecht et al. 1999; Spitz et al. 1996). There was only one replicated finding: MZ-MC twins were more similar than MZ-DC twins for the block design but not for vocabulary in children and adults (Spitz et al. 1996; Rose et al. 1981). One reason for the mixed findings in the literature likely is the small sample sizes used to investigate these effects. Nonetheless, there is evidence that chorionicity may have an effect on twin similarity for some cognitive measures, particularly during childhood. When effects were found, MC twins were generally more similar on the cognitive or personality assessment than DC twins were, suggesting that for some cognitive measures heritability estimates may be overestimated when not accounting for chorionicity.

We identified four studies that examined other behavioral phenotypes. For measures of temperament in very early childhood, MC twin similarity was equal to DC twin similarity (Chen et al. 1990; Riese 1999). Similarly, there was no chorionicity effect on twin similarity for prosocial behavior or Child Behavior Checklist (CBCL) total problems in childhood and adolescence (Hur 2007; Wichers et al. 2002). Thus, it is unlikely that chorionicity biases heritability estimates of toddler temperament and child and adolescent prosocial or problem behavior, although the studies were quite small and few in number.

## Discussion

We presented the state of the literature on twin chorionicity in relation to a series of human outcome traits, and addressed the question of to what extent chorionicity differences in MZ twins may influence heritability estimates. We found a large body of literature on the effects of chorionicity on health and behavioral outcomes and a much smaller, but notable body of literature (30 articles in total) that examined chorionicity in relation to twin similarity, which could be used to draw tentative conclusions about whether chorionicity may bias heritability estimates. With only three studies from Asian populations and no studies from African populations, we were unable to draw even tentative conclusions about whether potential chorionicity biases may differ in populations with different twinning rates and MZ-MC/MZ-DC/DZ-DC proportions.

Consistent with the theory that some chorionicity effects could lead to overestimation and others to underestimation of heritability, there were instances of each across the many phenotypes considered here. However, firm conclusions should not be drawn since some of the outcomes were only examined in one or few studies and often sample sizes were small. In this same issue, van Beijsterveldt et al. (2015), using a sample of over 9000 twin pairs, report on chorionicity and heritability estimates on 66 phenotypes, including weight, height, motor milestones, child problem behaviors, cognitive function, wellbeing and personality. For only a few traits, within-pair similarity differed between MC-MZ and DC-MZ pairs. For traits influenced by birth weight, such as weight in young children MC twins were more discordant for 5 out of 13 measures. For traits where blood supply is important, MC-MZ twins were more concordant than DC-MZ for 3 traits. van Beijsterveldt et al. conclude that “the influence on the MZ twin correlation of the intra-uterine prenatal environment, as measured by sharing a chorion type, is small and limited to a few phenotypes”.

In our review, we also see that the most robust findings for chorionicity biasing heritability estimates were for birth weight (Vlietinck et al. 1989; Gielen et al. 2008; Touwslager et al. 2010; see Buzzard et al. 1983 for trend effect). This may be due to differences in placental sharing and vascularization between MZ-MC co-twins, which would reduce MC twin similarity and subsequently underestimate heritability of BW (see Table 1). That chorionicity could lead to underestimates of heritability for birth weight is interesting because despite the low heritability estimates from twin studies for birth weight, recent genome-wide association studies for this phenotype yielded significant hits (Horikoshi et al. 2013; Freathy et al. 2010).

Chorionicity may continue to effect heritability estimates of anthropometric traits later in life, but here effects are attenuated and less consistent. For example, heritability of weight and BMI are likely to be underestimated in childhood and adolescence (Gutknecht et al. 1999; Spitz et al. 1996; Mukherjee et al. 2009), while findings for height are inconsistent (Hur and Shin 2008; Spitz et al. 1996; Gutknecht et al. 1999). By adulthood, chorionicity did not appear to bias heritability estimates for the majority of studied anthropomorphic measures (e.g., various obesity-related measures, lung measures, or conventional and ambulatory blood pressure (Loos et al. 2001a; van den Borst et al. 2012; Souren et al. 2007; Fagard et al. 2003), however, chorionicity had an effect on fasting fibrogen (Loos et al. 2001b). It is important to note that specific outcomes have not been studied systematically. Therefore, it is unclear to what extent chronicity affects specific anthropometric outcomes across development.

Similarly, the effect of chorionicity on cognitive and personality measures in childhood and adolescence was mixed, although when effects were found they pointed to overestimation of heritability estimates. In measures of early brain and cognitive development, chronicity appeared to play no role (Mukherjee et al. 2009; Melnick et al. 1980; Welch et al. 1978). Chorionicity also appeared to play no role in the twin similarity for trisomy 21, vaccination responses, handedness, toddler temperament, or child and adolescent prosocial or problem behavior. One study found evidence that heritability of was overestimated without accounting for chorionicity (Davis and Phelps 1995; Davis et al. 1995); however, this finding has yet to be replicated.

Taken together, chorionicity biases heritability estimates for some outcomes at some points in during development. It is unclear for which outcomes heritability estimates are likely to be biased in a meaningful or measurable way. This review suggests that outcomes that are related to birth weight are more likely to be influenced by chorionicity. There is also qualitative evidence to suggest that chorionicity effects on heritability may be relatively greater for early compared to later developmental outcomes, as was observed with anthropometric traits. With the exception of measures of birth weight and early growth, this review did not find evidence of any replicated effects of chorionicity on the heritability of human traits. Given the wide range of outcomes measured and small sample sizes it is unclear whether chronicity has a measurable effect on behavioral and cognitive measures. It thus would seem that concerns about heritability estimates based on the classical twin design, which relies on the equal environment assumption, are unwarranted when considering the prenatal environment.

### Electronic supplementary material

Below is the link to the electronic supplementary material.

Supplementary material 1 (DOCX 249 kb)
