# Supplementary material for: The Prenatal Environment in Twin Studies: A Review on Chorionicity
Source: Behav Genet. 2016 Mar 5;46:286–303. doi: 10.1007/s10519-016-9782-6 (PMC4858569; doi:10.1007/s10519-016-9782-6)
Supplement: Supplementary file 1 — Supplementary material 1 (DOCX 249 kb) [file 10519_2016_9782_MOESM1_ESM.docx]

Supplementary Table 1.

| **Construct** | **Reference** | **Effect** |
| --- | --- | --- |
| **Mortality** | Glinianaia et al., 2011 | MC > DC: still birth and neonatal mortality |
|  | Hack et al., 2008 | MC > DC: perinatal mortality and IUD after 32 weeks; DC > MC: GA BWT; MC > DC: BWD, necrotising enterocolitis after adjustment for age and bwt; trend for neuromorbidity |
|  | Hamilton et al., 1998 | MC > singletons: perinatal death; small MC = DC: perinatal mortality. DC = singletons: perinatal mortality |
|  | Ishii et al., 2014 | MC > DC: survival, overall and in % where all 3 made it in triplets with feto-fetal transfusion syndrome after fetoscopic laser photocoagulation |
|  | Kang et al., 2014 | MC > DC: IUFD in univariate but not multivariate logistic regressions |
|  | Kawaguchi et al., 2013 | MC > TC = DC: fetal death at > 22 weeks GA in triplets |
|  | Kilby et al., 1994 | MC > DC: sIUFD |
|  | Lee et al., 2008 | MC > DC: prevalence of stillbirth |
|  | Nobili et al., 2013 | MC > DC: procedure-related still birth and preterm delivery rate |
|  | Baghdadi et al., 2003 | MC = DC: fetal loss |
|  | Benson et al., 1993 | MC > DC: fetal loss |
|  | Bleker et al., 1997 | MC > DC: perinatal mortality |
|  | D'Antonio et al., 2013a | MC > DC early fetal loss <24weeks. MC = DC fetal loss from 24-34 weeks |
|  | D'Antonio et al., 2013b | MC = DC: fetal loss, median CRL discordance |
|  | Saito et al., 1999 | MC > DC: single fetal death |
|  | Lenis-Cordoba et al., 2013 | MC = DC: fetal loss and fetal loss <24 weeks |
|  | Kaufman et al., 2003 | MC > DC: fetal demise |
|  | McPherson et al., 2012 | MC > DC: any fetal demise, fetal demise of both, any nonanomalous fetal demise |
|  | Lynch et al., 2007 | MC > DC: perinatal loss |
|  | Mahony et al., 2011 | MC > DC: IUFD |
|  |  |  |
| **Diverse perinatal outcomes:**  **Morbidity**  **and mortality**  **Diverse perinatal outcomes:**  **Morbidity**  **and mortality**  **Diverse perinatal outcomes:**  **Morbidity**  **and mortality** | Adegbite 2005 AJOG | DC < TC: BWT, GA; TC < DC: risk of PROM in triplets |
|  | Fichera et al., 2009 | MC < DC: survival rate; MC = DC: GA, normal neurodevelopmental follow-ups |
|  | Fortin et al., 2005 | MC > DC: cerebral palsy, stillbirth rate |
|  | Acosta-Rojas et al., 2007 | MC < DC: GA, BW; MC > DC: NICU, fetal loss, neurological morbidity, respiratory problems, bronchopulmonary dysplasia, persistent ductus arteriosus |
|  | Carroll et al., 2005 | MZMC < MZDC: GA; MZMC < DZ: GA; proportion with BWD |
|  | D'Antonio et al., 2013c | MC = DC: median CRL discordance; MC > DC: mortality and SGA; proportion of BWD and estimated fetal weight |
|  | Dias et al., 2010 | MC = DC: CRL discordance; MC > DC: perinatal mortality |
|  | Dube et al., 2002 | MZMC > DZDC: Oligohydramnios, Polyhydramnios, perinatal death, IUGR; MZMC < DZDC: BWT |
|  | Hack et al., 2006 | MC < DC: BWT and GA; MC > DC perinatal mortality, preterm delivery, very preterm (<32 weeks) delivery, perinatal mortality in the presence of BWD |
|  | Hatkar et al., 1999 | MC > DC: perinatal mortality and low birth weight |
|  | Klatt et al., 2012 | MC = DC: single umbilical artery, C-section, SGA, BWD; DC > MC: GA, ART, BWT; MC > DC: miscarriage |
|  | Minakami et al., 1999 | DC > MC: GA, BWT for smaller and larger twin; MC > DC: VLBW (<1500 g), BWD |
|  | Oldenburg et al., 2012 | DC > MC: maternal age, ART conception, GA, BWT; MC > DC: pregnancy terminations, pregnancies with selective feticide, fetal loss<24weeks, pregnancies with fetal loss ≥24weeks, perinatal death, stillbirth |
|  | Quintana et al., 2013 | MC = DC: rate of C-section |
|  | Sebire et al., 1997 | MC > DC: fetal loss, BWT below <10% (for one fetus and for both fetuses), BWT below <5% (for one fetus and for both fetuses); DC > MC: BWT, GA; MC = DC: BWD, rate of premature birth (<32 or <37 weeks) |
|  | Shrim et al., 2010 | MC > DC: perinatal mortality, BW discordance (>20%), lower Apgar scores, congenital malformations, NICU admission, velamentous cord insertion; DC > MC: GA, BW, BW adjusted for GA |
|  | Tobe et al., 2010 | MC > DC: BWD, discharge mortality |
|  | Victoria et al., 2001 | MC > DC: BWD (>25%), preterm delivery (<30 weeks), NICU stay (>10 days; and >30 days); MC = DC: perinatal mortality rate. |
|  | Alam Machado 2009 | In BWT concordant twins: DC > MC: GA, BWT; MC > DC: length of stay; MC = DC in BWD group |
|  | Asztalos et al., 2001 | MC > DC: BWD, prematurity, low BWT, patent ductus arteriosus and sepsis, IUGR; MC = DC: death or severe neurodev morbidity |
|  | Bodeau-Livinec et al., 2013 | MC = DC: live birth/survivor at 5 years, CP, vision loss, severe auditory loss, MPC withough severe deficiency, overall deficiencies |
|  | Breathnach et al., 2012 | MC > DC: gestational age, low BWT, NICU, Morbidity |
|  | Ferreira et al., 2005 | MC > DC: pathology of amniotic fluid, discordant fetal growth, preterm delivery, low BWT, BWD, intraventricular hemorrhage, IUGR and mortality |
|  | Geipel et al., 2005 | MC = DC > TC: BWD, very premature, and IUFD |
|  | Manso et al., 2011 | MC > DC: preterm labor, oligohydramnios/hydramnios, discordant fetal growth, c- section, perinatal mortality, hyaline membrane disease, IVH, lower birth weight, and intrauterine growth restriction |
|  | Masheer et al., 2015 | MC > DC: lower birth weight, miscarriage, preterm birth, NICU admission, congenital anomalies, FGR |
|  | Pretorius et al., 1993 | MC > DC: pre- and perinatal mortality; sonographic errors |
|  | Spencer et al., 2009 | DC > TC: lower birth weight, lower gestational age, more time in NICU, culture proven sepsis, and require intubation |
|  | Leduc et al., 2005 | MC > DC: preterm deliveries between 30 and 34 weeks, < 10th percentile BWT, BWD (25%), NICU admission, and intraventricular hemorrage grade 3 and 4; MC = DC: preeclampsia, gestational diabetes, delivery method, mortality rate, sex, endotracheal intubation |
|  | Tagin et al., 2011 | MC > singletons, but DC = singletons: risk for mortality or severe disability |
|  | Suzuki and Matsuhashi 2007 | MC = DC: GA, BWT (of either twin) or umbilical arterial pH (of either twin) |
|  | S.T.O.R.C, 2012 | For Live births - MC < DC: BW, BWC, GA. For still births - MC = DC: BW, BWC, GA |
|  | Gielen et al., 2008 | DC > MC: BW, rate of primaparity; MC > DC: rate of very preterm (<32 weeks); rate of SGA, LBW, and VLBW; MC = DC: BWD (>25%) |
|  | Hjorto et al., 2014 | DC > MC: BW (first twin only), GA, pH <7.20, delivery interval; MC > DC: 1-min Apgar ≤7 |
|  | Honma et al., 1999 | Hemoglobin discordance predictive of postnatal disability in both MC and DC twins |
|  | Johansen et al., 2014 | MC twins: CRL discordance at 11-14 weeks predicted BW discordance and mean BW. DC twins: CRL discordance at 11-14 weeks predicted <34 week delivery, BW discordance and mean birth weight. |
|  | Vergani et al., 2004 | MC > DC: fetal death or adverse outcome. |
|  | Vergani et al., 2013 | MC < DC: GA; MC > DC: total adverse outcome, mild adverse outcome; MC = DC: severe adverse outcome. |
|  | Bajoria et al., 1999 | MC > DC: IUFD, anemia, intracranial lesions at birth, younger GA, same pattern in TTTS vs. no TTTS |
|  | Yu et al., 2002 | MC = DC: PI in the uterine arteries, GA, BMI, FGR, stillbirth, preterm delivery (<32 weeks) |
|  | Gezer et al., 2012 | MZMC > MZDC; MZMC > DZDC; MZDC = DZDC on all perinatal mortality and morbidity outcomes; MZ > DC: repiratory distress syndrome, intraventricular hemorrhage, NICU requrement, positive pressure ventialtion requirement |
|  | Morikawa et al., 2012 | MC < DC: maternal age, primiparity, ART conception, GA, rate of C-section, sum of twins BWT; MC > DC: still birth and early neonatal death |
|  |  |  |
| **Growth,**  **birth weight,**  **birth weight**  **discordance**  **Growth,**  **birth weight,**  **birth weight**  **discordance** | Senoo et al., 2000 | DC > MC: estimated fetal weight between 20-22 weeks gestation. No other significant differences emerged between MC and DC. |
|  | Taylor et al., 1998 | MC = DC: fetal growth velocity |
|  | Burgess et al., 2014 | MC > DC: low BWT, Csections; no real mortality |
|  | Gao et al., 2012 | MC and MZ both risk factors for sIGUR BUT MZMC = DZDC. |
|  | Gaziano et al., 1998 | MC > DC: probability of blood flow redistribution, lower GA at ultrasound, Umbilicial artery resistance, middle cerebral artery resistance, resistance index difference, and cerebral/placenta ratio |
|  | Gaziano et al., 2001 | MC < DC: lower CPR = (MCA/RI)/(UA/RI); RI difference, UA RI, and MCA RI (same as above) |
|  | Kent et al., 2012 | MC < DC: BWT; MC > DC: GA, composite perinatal morbidity (infarction, subchorial fibrin, retroplacental hematoma, abnormal villus maturation, and composite). MC = DC: BWD |
|  | Lee et al., 2010 | MZDC and MZMC > DZ twins on low BWT; MC = DC: low BWT; MC > DZDC: bronchopulmonary dysplasia |
|  | Pridjian et al., 1991 | MZMC > MZDC: BWD and polyhydramnios. Within MZMC, smaller twin had smaller brain weight and larger twin had larger heart weight |
|  | Canpolat et al., 2011 | MC = DC: IGF1 or BWT. Differences were in correlations: IGF1 and % BWT discordance were correlated in discordant but not concordant twins, and MC but not DC twins. |
|  | Cowans et al., 2013 | first trimester maternal serum placental growth factor: 41% higher in DC, 16% higher in MC compared with singletons. |
|  | Bhide et al., 2009 | MC > DC: fetal loss; severe BW discordance (≥25%). MC=DC BW discrepancy; CRL discrepancy at 12-13 weeks. |
|  | D'Antonio et al., 2013d | MC > DC: perinatal loss and BW discordance |
|  | Singh et al., 2014 | DC > MC: BWT |
|  | Ananth et al., 1998 | DC > MC: BWT adjusted for gestational age |
|  | Smith et al., 2001 | MC = DC: growth velocity across gestation |
|  | Sebire et al., 1998 | MC = DC: CRL discordance and BWD |
|  | Lamb et al., 2012 | DZ DC triplets > MZ MC triplets: BWT. BWD and GA did not differ based on zygosity/chorionicity classification. Chorionicity did not significantly predict BWT |
|  | Loos et al., 2001 | In males, DZ > MZMC: BWT. In males, DZ > MZDC, MZMC: GA. In females, DZ > MZMC: BWT. In females, DZ, MZDC > MZMC: GA. MZDC twins > DZ twins: larger trunk skinfolds. No other anthropometric measures were significant. In Males, MZDC < MZMC: Waist-to-hip ratio |
|  | Loos et al., 2005 | DZ > MZDC, MZMC: BWT and peripheral cord insertion in males and females. No differences in GA. DZ > MZMC: central cord insertion in males. |
|  | Luke et al., 2004 | MC < DC: Femur length<10th centile at 28 weeks gestation |
|  | Min 2004 | TC > MC or DC: BW, fetal growth references for triplet pregnancy provided in relation to chorionicity |
|  | Gonzalez-Quintero et al., 2003 | MC > DC, adjusted for parity: BW discordance |
|  | Papageorghiou et al., 2008 | DC > MC > TC: birth weight |
|  | Salomon et al., 2005 | MC = DC: CRL during 1st trimester |
|  | Lynch et al., 2003 | MC > DC: very low birth weight |
|  | Senoo et al., 2000 | MC > DC only in growth discordant twins, no difference in concordant MC vs DC twins |
|  | Snijder & Wladimiroff, 1998 | MC > DC: Abdominal circumference discordance, head circumference/abdominal circumference discordance |
|  |  |  |
| **Screening**  **Screening** | Sahota et al., 2009 | MC & DC > singletons: free β-hCG-MoM and PAPP-A MoM |
|  | Prats et al., 2012a | DC > MC: Β-hCG, PAPP-A. Trend for CRL. No differences for nuchal translucency thickness |
|  | Prats et al., 2012b | DC > MC: CRL, free-β-hCG. No differences for PAPP-A |
|  | Maymon et al., 2014 | MC = DC: nuchal translucency thickness |
|  | Monni et al., 2000 | MC = DC: nuchal translucency thickness; MC with TTTS demonstrated increased nuchal translucency thickness |
|  | Muller et al., 2003 | MC > DC: free beta-hCG. No differences for alpha-fetoprotein. |
|  | Gonce et al., 2010 | MC = DC: nuchal translucency thickness |
|  | Koster et al., 2010 | MC = DC for all markers |
|  | Cheng et al., 2010 | MC > DC: nuchal translucency (ignoring zygosity); MZMC = highest nuchal translucency thickness; MZDC slightly lower mean inter-twin nuchal translucency difference |
|  | Linskens et al., 2009 | MC < DC: all screening markers |
|  | Sebire et al., 1997 | MC with TTTS demonstrated increased nuchal translucency thickness |
|  | Sebire et al., 1996 | MC > DC: nuchal translucency thickness |
|  | Spencer et al., 2001 | MC = DC: b-hCG, PAPP-A, and nuchal translucency thickness |
|  | Sepulveda et al., 2009 | MC > DC: nuchal translucency thickness |
|  | Spencer et al., 2008 | DC > MC: PAPP-A following correction. MC = DC: beta-hCG |
|  | Sooranna et al., 2001 | MC concordant for growth = DC discordant for growth: delta fetal plasma leptin concentrations |
|  | Stiller et al., 1988 | DC > MC: amniotic fluid alpha-fetoprotein level discordance |
|  | Westwood et al., 2001 | MC = DC: IGF-I, IGF-II, total IGFBP-1 and lpIGFBP-1 concentrations; MC > DC: IGF-I in the IUGR twin within twin pairs discordant for growth; MC < DC: IGF-II in the IUGR twin within twin pairs discordant for growth. |
|  | Hershkovitz et al., 2005 | MC > DC: MShCG >3.0 MoM and mean MShCG |
|  | Phung et al., 2002 | MC > DC: chorioamnionitis, chorioamnionitis and funisitis |
|  |  |  |
| **Congenital**  **defects/**  **malformations/**  **anomalies** | Herskind et al., 2013 | MC > singletons: congenital heart defects. Also, MZ=DZ, DZ > singletons, unknown zygosity > singletons |
|  | Hajdu et al., 2006 | MZ > DZ or DC (with unknown zygosity): heart disease |
|  | Corney et al., 1983 | MC = DC: congenital malformations |
|  | Glinianaia et al., 2008 | MC > DC prevalence; twins > singletons: congenital anomalies (cardiovascular, CNS, genito-urinary, chromosomal, musculoskeletal, and others) |
|  | Gupta et al., 2010 | MC = DC; MZ = DZ: congenital malformations |
|  | Gul et al., 2005 | MC = DC: fetal anomaly discordance (GA, BWT, perinatal survival in twins with vs without anamolies |
|  | Harper et al., 2012 | MC = DC: anomaly status |
|  |  |  |
| **Misc. perinatal outcomes** | Visentin et al., 2013 | MC = DC: Aorta intima thickness |
|  | Maiz et al., 2009 | MC > DC for reverse a-wave. DC > MC: normal pregnancy outcomes |
|  | De Silva et al., 1992 | MC < DC: umbilical cord length and width |
|  | Eberle et al., 1993 | DC but not MC: number of placental lesions related to BWD |
|  | Guilherme et al., 2009 | TC > MC & DC: frequency following ART compared to spontaneous conception |
|  |  |  |
| **Brain outcomes** | Adegbite 2005 EJOGRB | MC > DC: cerebral white matter lesions. Discordant weight, TTTS, sIUFD also risk factors |
|  | Bejar et al., 1990 | incidence MC > DC: incidence of antenatal necrosis of cerebral white matter |
|  | van Steenis et al., 2014 | MC > DC: required blood transfusion at birth; DC > MC: Hemoglobin level at birth. No differences for other lab, clinical neurologic indices of perinatal asphyxia and cerebral injury |
|  |  |  |
| **Cognitive**  **outcomes** | Einaudi et al., 2008 | MC > DC: pathological nonverbal performances, learning disabilities |
|  | Welch et al., 1978 | MZMC = MZDC: BWT, Bayley mental development index; MZMC > MZDC: BWD |
|  | Steingass et al., 2013 | MC > DC: SGA; MC = DC: cerebral palsy, neurodevelopmental impairment, Bayley Mental Development Index |
|  | Hack et al., 2009 | MC = DC: cerebral palsy and neurodevelopmental outcomes at 22 months (corrected age) |
|  | Livinec et al., 2005 | MC > DC but association not significant after adjustment for pregnancy complication, sex, gestational age, and prenatal corticotherapy |
|  | Burguet et al., 1999 | MC > DC: cerebral palsy |
|  | Burguet et al., 1995 | MC > DC: neurodevelopmental disability |
|  |  |  |
| **Misc.**  **psychiatric/**  **behavioral**  **outcomes** | Piontelli et al., 1997 | Intrapair stimulation was observed in MC twins before 11+ weeks GA and from 12+weeks GA for DC twins |
|  | Derom et al. 1996 | Twins are more frequently left handed, not related to zygosity or chorionicity |
|  | Natalucci et al., 2012 | DC (i.e., 2 of 3 triplets share a placenta) predicts lower self- and parent reported health related quality of life |

Note. Described effects are not comprehensive. MC = monochorionic. DC = dichorionic. ART = artificial reproductive technologies. BWT = birth weight. BWD = birth weight discordance. IUFD = intrauterine fetal death; sIUFD = selective intrauterine fetal death. IUGR = intrauterine growth retardation. GA = gestational age. CRL = crown-rump length. VLBW = very low birth weight. TTTS = twin-to-twin transfusion syndrome, MShCG = Maternal Serum Human Chorionic Gonadotrophin, MoM = multiples of the median, IGF-I = insulin-like growth factor I, IGF-II = insulin-like growth factor II, IGFBP-1 = insulin-like growth factor-binding protein, lpIGFBP-1 = less-phosphorylated insulin-like growth factor-binding protein, b-hCG = beta-human chorionic gonadotropin, PAPP-A = Pregnancy-associated plasma protein A, Hcg = human chorionic gonadotropin, FGR = fetal growth restriction, SGA = small for gestational age

(STORK), S. T. O. R. C. (2012). Prospective risk of late stillbirth in monochorionic twins: a regional cohort study. *Ultrasound Obstet Gynecol, 39*(5), 500-504, doi:10.1002/uog.11110.

Acosta-Rojas, R., Becker, J., Munoz-Abellana, B., Ruiz, C., Carreras, E., & Gratacos, E. (2007). Twin chorionicity and the risk of adverse perinatal outcome. *Int J Gynaecol Obstet, 96*(2), 98-102, doi:10.1016/j.ijgo.2006.11.002.

Adegbite, A. L., Castille, S., Ward, S., & Bajoria, R. (2005a). Prevalence of cranial scan abnormalities in preterm twins in relation to chorionicity and discordant birth weight. *Eur J Obstet Gynecol Reprod Biol, 119*(1), 47-55, doi:10.1016/j.ejogrb.2004.06.016.

Adegbite, A. L., Ward, S. B., & Bajoria, R. (2005b). Perinatal outcome of spontaneously conceived triplet pregnancies in relation to chorionicity. *Am J Obstet Gynecol, 193*(4), 1463-1471, doi:10.1016/j.ajog.2005.02.098.

Alam Machado, R. D. C., Brizot, M. D. L., Liao, A. W., Krebs, V. L. J., & Zugaib, M. (2009). Early neonatal morbidity and mortality in growth-discordant twins. *Acta Obstet Gynecol Scand, 88*(2), 167-171, doi:<http://dx.doi.org/10.1080/00016340802649808>.

Ananth, C. V., Vintzileos, A. M., Shen-Schwarz, S., Smulian, J. C., & Lai, Y. L. (1998). Standards of birth weight in twin gestations stratified by placental chorionicity. *Obstet Gynecol, 91*(6), 917-924.

Asztalos, E., Barrett, J. F., Lacy, M., & Luther, M. (2001). Evaluating 2 year outcome in twins < or = 30 weeks gestation at birth: a regional perinatal unit's experience. *Twin Res, 4*(6), 431-438.

Baghdadi, S., Gee, H., Whittle, M. J., & Khan, K. S. (2003). Twin pregnancy outcome and chorionicity. *Acta Obstet Gynecol Scand, 82*(1), 18-21.

Bajoria, R., Wee, L. Y., Anwar, S., & Ward, S. (1999). Outcome of twin pregnancies complicated by single intrauterine death in relation to vascular anatomy of the monochorionic placenta. *Hum Reprod, 14*(8), 2124-2130.

Bejar, R., Vigliocco, G., Gramajo, H., Solana, C., Benirschke, K., Berry, C., et al. (1990). Antenatal origin of neurologic damage in newborn infants. II. Multiple gestations. *Am J Obstet Gynecol, 162*(5), 1230-1236.

Benson, C. B., Doubilet, P. M., & Laks, M. P. (1993). Outcome of twin gestations following sonographic demonstration of two heart beats in the first trimester. *Ultrasound Obstet Gynecol, 3*(5), 343-345, doi:10.1046/j.1469-0705.1993.03050343.x.

Bhide, A., Sankaran, S., Sairam, S., Papageorghiou, A. T., & Thilaganathan, B. (2009). Relationship of intertwin crown-rump length discrepancy to chorionicity, fetal demise and birth-weight discordance. *Ultrasound Obstet Gynecol, 34*(2), 131-135, doi:10.1002/uog.6396.

Bleker, O. P., & Oosting, H. (1997). Term and postterm twin gestations. Placental cause of perinatal mortality. *J Reprod Med, 42*(11), 715-718.

Bodeau-Livinec, F., Zeitlin, J., Blondel, B., Arnaud, C., Fresson, J., Burguet, A., et al. (2013). Do very preterm twins and singletons differ in their neurodevelopment at 5 years of age? *Arch Dis Child Fetal Neonatal Ed, 98*(6), F480-487, doi:10.1136/archdischild-2013-303737.

Breathnach, F. M., McAuliffe, F. M., Geary, M., Daly, S., Higgins, J. R., Dornan, J., et al. (2012). Optimum timing for planned delivery of uncomplicated monochorionic and dichorionic twin pregnancies. *Obstet Gynecol, 119*(1), 50-59, doi:10.1097/AOG.0b013e31823d7b06.

Burgess, J. L., Unal, E. R., Nietert, P. J., & Newman, R. B. (2014). Risk of late-preterm stillbirth and neonatal morbidity for monochorionic and dichorionic twins. *Am J Obstet Gynecol, 210*(6), 578.e571-579, doi:10.1016/j.ajog.2014.03.003.

Burguet, A., Menget, A., Monnet, E., Allemand, H., Gasca-Avanzi, A., Laithier, V., et al. (1995). [Neurologic development in premature infants under 33 weeks of gestational age: determination of risk of neurological abnormalities in a prospective regional survey with a control group]. *Arch Pediatr, 2*(12), 1157-1165.

Burguet, A., Monnet, E., Pauchard, J. Y., Roth, P., Fromentin, C., Dalphin, M. L., et al. (1999). Some risk factors for cerebral palsy in very premature infants: importance of premature rupture of membranes and monochorionic twin placentation. *Biol Neonate, 75*(3), 177-186, doi:14094.

Canpolat, F. E., Cekmez, F., Sarici, S. U., Korkmaz, A., & Yurdakok, M. (2011). Insulin-like growth factor-1 levels in twins and its correlation with discordance. *Twin Res Hum Genet, 14*(1), 94-97, doi:10.1375/twin.14.1.94.

Carroll, S. G., Tyfield, L., Reeve, L., Porter, H., Soothill, P., & Kyle, P. M. (2005). Is zygosity or chorionicity the main determinant of fetal outcome in twin pregnancies? *Am J Obstet Gynecol, 193*(3 Pt 1), 757-761, doi:10.1016/j.ajog.2005.01.024.

Cheng, P. J., Huang, S. Y., Shaw, S. W., Hsiao, C. H., Kao, C. C., Chueh, H. Y., et al. (2010). Difference in nuchal translucency between monozygotic and dizygotic spontaneously conceived twins. *Prenat Diagn, 30*(3), 247-250, doi:10.1002/pd.2450.

Corney, G., MacGillivray, I., & Campbell, D. M. (1983). Congenital anomalies in twins in Aberdeen and Northeast Scotland. *Acta Genet Med Gemellol (Roma), 32*(1), 31-35.

Cowans, N. J., & Spencer, K. (2013). First trimester maternal serum placental growth factor levels in twin pregnancies. *Prenat Diagn, 33*(13), 1260-1263, doi:10.1002/pd.4243.

D'Antonio, F., Khalil, A., Dias, T., & Thilaganathan, B. (2013a). Crown-rump length discordance and adverse perinatal outcome in twins: analysis of the Southwest Thames Obstetric Research Collaborative (STORK) multiple pregnancy cohort. *Ultrasound Obstet Gynecol, 41*(6), 621-626, doi:10.1002/uog.12430.

D'Antonio, F., Khalil, A., Dias, T., & Thilaganathan, B. (2013b). Early fetal loss in monochorionic and dichorionic twin pregnancies: analysis of the Southwest Thames Obstetric Research Collaborative (STORK) multiple pregnancy cohort. *Ultrasound Obstet Gynecol, 41*(6), 632-636, doi:10.1002/uog.12363.

D'Antonio, F., Khalil, A., Dias, T., & Thilaganathan, B. (2013c). Weight discordance and perinatal mortality in twins: analysis of the Southwest Thames Obstetric Research Collaborative (STORK) multiple pregnancy cohort. *Ultrasound Obstet Gynecol, 41*(6), 643-648, doi:10.1002/uog.12412.

D'Antonio, F., Khalil, A., Mantovani, E., & Thilaganathan, B. (2013d). Embryonic growth discordance and early fetal loss: the STORK multiple pregnancy cohort and systematic review. *Hum Reprod, 28*(10), 2621-2627, doi:10.1093/humrep/det277.

De Silva, N. (1992). Zygosity and umbilical cord length. [Conference Paper]. *Journal of Reproductive Medicine for the Obstetrician and Gynecologist, 37*(10), 850-852.

Derom, C., Thiery, E., Vlietinck, R., & Loos, R. (1996). Handedness in twins according to zygosity and chorion type: A preliminary report. *Behav Genet, 26*(4), 407-408, doi:<http://dx.doi.org/10.1007/BF02359484>.

Dias, T., Bhide, A., & Thilaganathan, B. (2010). Early pregnancy growth and pregnancy outcome in twin pregnancies. *Ceylon Med J, 55*(3), 80-84.

Dube, J., Dodds, L., & Armson, B. A. (2002). Does chorionicity or zygosity predict adverse perinatal outcomes in twins? *Am J Obstet Gynecol, 186*(3), 579-583.

Eberle, A. M., Levesque, D., Vintzileos, A. M., Egan, J. F., Tsapanos, V., & Salafia, C. M. (1993). Placental pathology in discordant twins. *Am J Obstet Gynecol, 169*(4), 931-935.

Einaudi, M. A., Busuttil, M., Monnier, A. S., Chanus, I., Palix, C., & Gire, C. (2008). Neuropsychological screening of a group of preterm twins: comparison with singletons. *Childs Nerv Syst, 24*(2), 225-230, doi:10.1007/s00381-007-0422-6.

Ferreira, I., Laureano, C., Branco, M., Nordeste, A., Fonseca, M., Pinheiro, A., et al. (2005). Chorionicity and adverse perinatal outcome. [Portuguese]. *Acta Med Port, 18*(3), 183-188.

Fichera, A., Zambolo, C., Accorsi, P., Martelli, P., Ambrosi, C., & Frusca, T. (2009). Perinatal outcome and neurological follow up of the cotwins in twin pregnancies complicated by single intrauterine death. *Eur J Obstet Gynecol Reprod Biol, 147*(1), 37-40, doi:10.1016/j.ejogrb.2009.07.002.

Fortin, A., Rajguru, M., Madelenat, P., & Mahieu-Caputo, D. (2005). [Neurological outcome of children from twin pregnancies]. *Gynecol Obstet Fertil, 33*(9), 563-569, doi:10.1016/j.gyobfe.2005.07.019.

Gao, Y., He, Z., Luo, Y., Sun, H., Huang, L., Li, M., et al. (2012). Selective and non-selective intrauterine growth restriction in twin pregnancies: high-risk factors and perinatal outcome. *Arch Gynecol Obstet, 285*(4), 973-978, doi:10.1007/s00404-011-2119-z.

Gaziano, E., Gaziano, C., & Brandt, D. (1998). Doppler velocimetry determined redistribution of fetal blood flow: correlation with growth restriction in diamniotic monochorionic and dizygotic twins. *Am J Obstet Gynecol, 178*(6), 1359-1367.

Gaziano, E. P., Gaziano, C., Terrell, C. A., & Hoekstra, R. E. (2001). The cerebroplacental Doppler ratio and neonatal outcome in diamnionic monochorionic and dichorionic twins. *J Matern Fetal Med, 10*(6), 371-375.

Geipel, A., Berg, C., Katalinic, A., Plath, H., Hansmann, M., Germer, U., et al. (2005). Prenatal diagnosis and obstetric outcomes in triplet pregnancies in relation to chorionicity. *Bjog, 112*(5), 554-558, doi:10.1111/j.1471-0528.2005.00627.x.

Gezer, A., Rashidova, M., Guralp, O., & Ocer, F. (2012). Perinatal mortality and morbidity in twin pregnancies: the relation between chorionicity and gestational age at birth. *Arch Gynecol Obstet, 285*(2), 353-360, doi:10.1007/s00404-011-1973-z.

Gielen, M., Lindsey, P. J., Derom, C., Loos, R. J., Souren, N. Y., Paulussen, A. D., et al. (2008). Twin-specific intrauterine 'growth' charts based on cross-sectional birthweight data. *Twin Res Hum Genet, 11*(2), 224-235, doi:10.1375/twin.11.2.224.

Glinianaia, S. V., Obeysekera, M. A., Sturgiss, S., & Bell, R. (2011). Stillbirth and neonatal mortality in monochorionic and dichorionic twins: a population-based study. *Hum Reprod, 26*(9), 2549-2557, doi:10.1093/humrep/der213.

Glinianaia, S. V., Rankin, J., & Wright, C. (2008). Congenital anomalies in twins: a register-based study. *Hum Reprod, 23*(6), 1306-1311, doi:10.1093/humrep/den104.

Gonce, A., Borrell, A., Meler, E., Arigita, M., Martinez, J. M., Botet, F., et al. (2010). Prevalence and perinatal outcome of dichorionic and monochorionic twins with nuchal translucency above the 99(th) percentile and normal karyotype. *Ultrasound Obstet Gynecol, 35*(1), 14-18, doi:10.1002/uog.7498.

Gonzalez-Quintero, V. H., Luke, B., O'Sullivan M, J., Misiunas, R., Anderson, E., Nugent, C., et al. (2003). Antenatal factors associated with significant birth weight discordancy in twin gestations. *Am J Obstet Gynecol, 189*(3), 813-817.

Guilherme, R., Le Ray, C., Vuillard, E., Garel, C., Delezoide, A. L., Oury, J. F., et al. (2009). Ultrasound assessment of the prognosis in triplet pregnancies. *Acta Obstet Gynecol Scand, 88*(4), 386-390, doi:10.1080/00016340902792433.

Gul, A., Cebeci, A., Aslan, H., Polat, I., Sozen, I., & Ceylan, Y. (2005). Perinatal outcomes of twin pregnancies discordant for major fetal anomalies. *Fetal Diagn Ther, 20*(4), 244-248, doi:10.1159/000085078.

Gupta, P., Faridi, M. M., & Dev, G. (2010). Congenital malformations in twins: effect of chorionicity and zygosity. *Indian Pediatr, 47*(4), 343-344.

Hack, K. E., Derks, J. B., de Visser, V. L., Elias, S. G., & Visser, G. H. (2006). The natural course of monochorionic and dichorionic twin pregnancies: a historical cohort. *Twin Res Hum Genet, 9*(3), 450-455, doi:10.1375/183242706777591281.

Hack, K. E., Derks, J. B., Elias, S. G., Franx, A., Roos, E. J., Voerman, S. K., et al. (2008). Increased perinatal mortality and morbidity in monochorionic versus dichorionic twin pregnancies: clinical implications of a large Dutch cohort study. *Bjog, 115*(1), 58-67, doi:10.1111/j.1471-0528.2007.01556.x.

Hack, K. E., Koopman-Esseboom, C., Derks, J. B., Elias, S. G., de Kleine, M. J., Baerts, W., et al. (2009). Long-term neurodevelopmental outcome of monochorionic and matched dichorionic twins. *PLoS One, 4*(8), e6815, doi:10.1371/journal.pone.0006815.

Hajdu, J., Beke, A., Marton, T., Hruby, E., Pete, B., & Papp, Z. (2006). Congenital heart diseases in twin pregnancies. *Fetal Diagn Ther, 21*(2), 198-203, doi:10.1159/000089303.

Hamilton, E. F., Platt, R. W., Morin, L., Usher, R., & Kramer, M. (1998). How small is too small in a twin pregnancy? *Am J Obstet Gynecol, 179*(3 Pt 1), 682-685.

Harper, L. M., Odibo, A. O., Roehl, K. A., Longman, R. E., MacOnes, G. A., & Cahill, A. G. (2012). Risk of preterm delivery and growth restriction in twins discordant for structural anomalies. *Am J Obstet Gynecol, 206*(1), 70.e71-70.e75, doi:<http://dx.doi.org/10.1016/j.ajog.2011.07.025>.

Hatkar, P. A., & Bhide, A. G. (1999). Perinatal outcome of twins in relation to chorionicity. *J Postgrad Med, 45*(2), 33-37.

Hershkovitz, R., Bar, G., Erez, O., Smolin, A., Sheiner, E., Mishori-Dery, A., et al. (2005). Increased maternal serum human chorionic gonadotropin concentrations are an independent risk factor for SGA in dichorionic twin gestations. *J Matern Fetal Neonatal Med, 18*(2), 117-122, doi:10.1080/14767050500199228.

Herskind, A. M., Almind Pedersen, D., & Christensen, K. (2013). Increased prevalence of congenital heart defects in monozygotic and dizygotic twins. *Circulation, 128*(11), 1182-1188, doi:10.1161/circulationaha.113.002453.

Hjorto, S., Nickelsen, C., Petersen, J., & Secher, N. J. (2014). The effect of chorionicity and twin-to-twin delivery time interval on short-term outcome of the second twin. *J Matern Fetal Neonatal Med, 27*(1), 42-47, doi:10.3109/14767058.2013.799657.

Honma, Y., Minakami, H., Eguchi, Y., Uchida, A., Izumi, A., & Sato, I. (1999). Relation between hemoglobin discordance and adverse outcome in monochorionic twins. *Acta Obstet Gynecol Scand, 78*(3), 207-211.

Ishii, K., Nakata, M., Wada, S., Hayashi, S., Murakoshi, T., & Sago, H. (2014). Perinatal outcome after laser surgery for triplet gestations with feto-fetal transfusion syndrome. *Prenat Diagn, 34*(8), 734-738, doi:<http://dx.doi.org/10.1002/pd.4357>.

Johansen, M. L., Oldenburg, A., Rosthoj, S., Cohn Maxild, J., Rode, L., & Tabor, A. (2014). Crown-rump length discordance in the first trimester: a predictor of adverse outcome in twin pregnancies? *Ultrasound Obstet Gynecol, 43*(3), 277-283, doi:10.1002/uog.12534.

Kang, H. J., Liao, A. W., Brizot, M. L., Francisco, R. P., Krebs, V. L., & Zugaib, M. (2014). Prediction of intrauterine death and severe preterm delivery in twin pregnancies discordant for major fetal abnormality. *Eur J Obstet Gynecol Reprod Biol, 175*, 115-118, doi:10.1016/j.ejogrb.2014.01.003.

Kaufman, H. K., Hume, R. F., Jr., Calhoun, B. C., Carlson, N., Yorke, V., Elliott, D., et al. (2003). Natural history of twin gestation complicated by in utero fetal demise: associations of chorionicity, prematurity, and maternal morbidity. *Fetal Diagn Ther, 18*(6), 442-446, doi:73140.

Kawaguchi, H., Ishii, K., Yamamoto, R., Hayashi, S., & Mitsuda, N. (2013). Perinatal death of triplet pregnancies by chorionicity. *Am J Obstet Gynecol, 209*(1), 36.e31-37, doi:10.1016/j.ajog.2013.03.003.

Kent, E. M., Breathnach, F. M., Gillan, J. E., McAuliffe, F. M., Geary, M. P., Daly, S., et al. (2012). Placental pathology, birthweight discordance, and growth restriction in twin pregnancy: results of the ESPRiT Study. *Am J Obstet Gynecol, 207*(3), 220.e221-225, doi:10.1016/j.ajog.2012.06.022.

Kilby, M. D., Govind, A., & O'Brien, P. M. (1994). Outcome of twin pregnancies complicated by a single intrauterine death: a comparison with viable twin pregnancies. *Obstet Gynecol, 84*(1), 107-109.

Klatt, J., Kuhn, A., Baumann, M., & Raio, L. (2012). Single umbilical artery in twin pregnancies. *Ultrasound Obstet Gynecol, 39*(5), 505-509, doi:10.1002/uog.9085.

Koster, M. P., Wortelboer, E. J., Stoutenbeek, P., Visser, G. H., & Schielen, P. C. (2010). Distributions of current and new first-trimester Down syndrome screening markers in twin pregnancies. *Prenat Diagn, 30*(5), 413-417, doi:10.1002/pd.2483.

Lamb, D. J., Vink, J. M., Middeldorp, C. M., van Beijsterveldt, C. E., Haak, M. C., Overbeek, L. I., et al. (2012). Effects of chorionicity and zygosity on triplet birth weight. *Twin Res Hum Genet, 15*(2), 149-157, doi:10.1375/twin.15.2.149.

Leduc, L., Takser, L., & Rinfret, D. (2005). Persistance of adverse obstetric and neonatal outcomes in monochorionic twins after exclusion of disorders unique to monochorionic placentation. *Am J Obstet Gynecol, 193*(5), 1670-1675, doi:10.1016/j.ajog.2005.04.007.

Lee, K. A., Oh, K. J., Lee, S. M., Kim, A., & Jun, J. K. (2010). The frequency and clinical significance of twin gestations according to zygosity and chorionicity. *Twin Res Hum Genet, 13*(6), 609-619, doi:10.1375/twin.13.6.609.

Lee, Y. M., Wylie, B. J., Simpson, L. L., & D'Alton, M. E. (2008). Twin chorionicity and the risk of stillbirth. *Obstet Gynecol, 111*(2 Pt 1), 301-308, doi:10.1097/AOG.0b013e318160d65d.

Lenis-Cordoba, N., Sanchez, M. A., Bello-Munoz, J. C., Sagala-Martinez, J., Campos, N., Carreras-Moratonas, E., et al. (2013). Amniocentesis and the risk of second trimester fetal loss in twin pregnancies: results from a prospective observational study. *J Matern Fetal Neonatal Med, 26*(15), 1537-1541, doi:10.3109/14767058.2013.791271.

Linskens, I. H., Spreeuwenberg, M. D., Blankenstein, M. A., & van Vugt, J. M. (2009). Early first-trimester free beta-hCG and PAPP-A serum distributions in monochorionic and dichorionic twins. *Prenat Diagn, 29*(1), 74-78, doi:10.1002/pd.2184.

Livinec, F., Ancel, P. Y., Marret, S., Arnaud, C., Fresson, J., Pierrat, V., et al. (2005). Prenatal risk factors for cerebral palsy in very preterm singletons and twins. *Obstet Gynecol, 105*(6), 1341-1347, doi:10.1097/01.AOG.0000161375.55172.3f.

Loos, R. J., Derom, C., Derom, R., & Vlietinck, R. (2001). Birthweight in liveborn twins: the influence of the umbilical cord insertion and fusion of placentas. *Bjog, 108*(9), 943-948.

Loos, R. J., Derom, C., Derom, R., & Vlietinck, R. (2005). Determinants of birthweight and intrauterine growth in liveborn twins. *Paediatr Perinat Epidemiol, 19 Suppl 1*, 15-22, doi:10.1111/j.1365-3016.2005.00611.x.

Luke, B., Brown, M. B., Hediger, M. L., Nugent, C., Misiunas, R. B., & Anderson, E. (2004). Fetal phenotypes and neonatal and early childhood outcomes in twins. *Am J Obstet Gynecol, 191*(4), 1270-1276, doi:10.1016/j.ajog.2004.03.006.

Lynch, A., McDuffie, R., Jr., Lyons, E., Chase, M., & Orleans, M. (2007). Perinatal loss among twins. *Perm J, 11*(1), 7-12.

Lynch, A., McDuffie, R., Stephens, J., Murphy, J., Faber, K., & Orleans, M. (2003). The contribution of assisted conception, chorionicity and other risk factors to very low birthweight in a twin cohort. *Bjog, 110*(4), 405-410.

Mahony, R., Mulcahy, C., McAuliffe, F., Herlihy, C. O., Carroll, S., & Foley, M. E. (2011). Fetal death in twins. *Acta Obstet Gynecol Scand, 90*(11), 1274-1280, doi:10.1111/j.1600-0412.2011.01239.x.

Maiz, N., Staboulidou, I., Leal, A. M., Minekawa, R., & Nicolaides, K. H. (2009). Ductus venosus Doppler at 11 to 13 weeks of gestation in the prediction of outcome in twin pregnancies. *Obstet Gynecol, 113*(4), 860-865, doi:10.1097/AOG.0b013e31819c9f66.

Manso, P., Vaz, A., Taborda, A., & Silva, I. S. (2011). Chorionicity and perinatal complications in twin pregnancy a 10 years case series. *Acta Med Port, 24*(5), 695-698.

Masheer, S., Maheen, H., & Munim, S. (2015). Perinatal outcome of twin pregnancies according to chorionicity: an observational study from tertiary care hospital. *J Matern Fetal Neonatal Med, 28*(1), 23-25, doi:10.3109/14767058.2014.899576.

Maymon, R., Cuckle, H., Svirsky, R., Sheena, L., Melcer, Y., Rozen, H., et al. (2014). Nuchal translucency in twins according to mode of assisted conception and chorionicity. *Ultrasound Obstet Gynecol, 44*(1), 38-43, doi:10.1002/uog.13278.

McPherson, J. A., Odibo, A. O., Shanks, A. L., Roehl, K. A., Macones, G. A., & Cahill, A. G. (2012). Impact of chorionicity on risk and timing of intrauterine fetal demise in twin pregnancies. *Am J Obstet Gynecol, 207*(3), 190.e191-196, doi:10.1016/j.ajog.2012.07.031.

Min, S. J., Luke, B., Min, L., Misiunas, R., Nugent, C., Van de Ven, C., et al. (2004). Birth weight references for triplets. *Am J Obstet Gynecol, 191*(3), 809-814, doi:10.1016/j.ajog.2004.01.052.

Minakami, H., Honma, Y., Matsubara, S., Uchida, A., Shiraishi, H., & Sato, I. (1999). Effects of placental chorionicity on outcome in twin pregnancies. A cohort study. *J Reprod Med, 44*(7), 595-600.

Monni, G., Zoppi, M. A., Ibba, R. M., Putzolu, M., & Floris, M. (2000). Nuchal translucency in multiple pregnancies. *Croat Med J, 41*(3), 266-269.

Morikawa, M., Yamada, T., Yamada, T., Sato, S., Cho, K., & Minakami, H. (2012). Prospective risk of stillbirth: monochorionic diamniotic twins vs. dichorionic twins. *J Perinat Med, 40*(3), 245-249, doi:10.1515/jpm-2011-0205.

Muller, F., Dreux, S., Dupoizat, H., Uzan, S., Dubin, M. F., Oury, J. F., et al. (2003). Second-trimester Down syndrome maternal serum screening in twin pregnancies: impact of chorionicity. *Prenat Diagn, 23*(4), 331-335, doi:10.1002/pd.594.

Natalucci, G., Iten, M., Hofmann, J., Bucher, H. U., Arlettaz, R., Molinari, L., et al. (2012). Health-related quality of life and behavior of triplets at adolescent age. *J Pediatr, 161*(3), 495-500.e491, doi:10.1016/j.jpeds.2012.03.001.

Nobili, E., Paramasivam, G., & Kumar, S. (2013). Outcome following selective fetal reduction in monochorionic and dichorionic twin pregnancies discordant for structural, chromosomal and genetic disorders. *Aust N Z J Obstet Gynaecol, 53*(2), 114-118, doi:10.1111/ajo.12071.

Oldenburg, A., Rode, L., Bodker, B., Ersbak, V., Holmskov, A., Jorgensen, F. S., et al. (2012). Influence of chorionicity on perinatal outcome in a large cohort of Danish twin pregnancies. *Ultrasound Obstet Gynecol, 39*(1), 69-74, doi:10.1002/uog.10057.

Papageorghiou, A. T., Bakoulas, V., Sebire, N. J., & Nicolaides, K. H. (2008). Intrauterine growth in multiple pregnancies in relation to fetal number, chorionicity and gestational age. *Ultrasound Obstet Gynecol, 32*(7), 890-893, doi:10.1002/uog.6140.

Phung, D. T., Blickstein, I., Goldman, R. D., Machin, G. A., LoSasso, R. D., & Keith, L. G. (2002). The Northwestern Twin Chorionicity Study: I. Discordant inflammatory findings that are related to chorionicity in presenting versus nonpresenting twins. *Am J Obstet Gynecol, 186*(5), 1041-1045.

Piontelli, A., Bocconi, L., Kustermann, A., Tassis, B., Zoppini, C., & Nicolini, U. (1997). Patterns of evoked behaviour in twin pregnancies during the first 22 weeks of gestation. *Early Hum Dev, 50*(1), 39-45.

Prats, P., Rodriguez, I., Comas, C., & Puerto, B. (2012a). First trimester risk assessment for trisomy 21 in twin pregnancies combining nuchal translucency and first trimester biochemical markers. *Prenat Diagn, 32*(10), 927-932, doi:10.1002/pd.3934.

Prats, P., Rodriguez, I., Nicolau, J., & Comas, C. (2012b). Early first-trimester free-beta-hCG and PAPP-A serum distributions in monochorionic and dichorionic twins. *Prenat Diagn, 32*(1), 64-69, doi:10.1002/pd.2902.

Pretorius, D. H., Budorick, N. E., Scioscia, A. L., Krabbe, J. K., Ko, S., & Myhre, C. M. (1993). Twin pregnancies in the second trimester in women in an alpha-fetoprotein screening program: sonographic evaluation and outcome. *AJR Am J Roentgenol, 161*(5), 1007-1013, doi:10.2214/ajr.161.5.7506005.

Pridjian, G., Nugent, C. E., & Barr, M., Jr. (1991). Twin gestation: influence of placentation on fetal growth. *Am J Obstet Gynecol, 165*(5 Pt 1), 1394-1401.

Quintana, E., Burgos, J., Eguiguren, N., Melchor, J. C., Fernandez-Llebrez, L., & Martinez-Astorquiza, T. (2013). Influence of chorionicity in intra-partum management of twin deliveries. *J Matern Fetal Neonatal Med, 26*(4), 407-411, doi:10.3109/14767058.2012.733752.

Sahota, D. S., Leung, T. Y., Fung, T. Y., Chan, L. W., Law, L. W., & Lau, T. K. (2009). Medians and correction factors for biochemical and ultrasound markers in Chinese women undergoing first-trimester screening for trisomy 21. *Ultrasound Obstet Gynecol, 33*(4), 387-393, doi:10.1002/uog.6340.

Saito, K., Ohtsu, Y., Amano, K., & Nishijima, M. (1999). Perinatal outcome and management of single fetal death in twin pregnancy: a case series and review. *J Perinat Med, 27*(6), 473-477, doi:10.1515/jpm.1999.063.

Salomon, L. J., Cavicchioni, O., Bernard, J. P., Duyme, M., & Ville, Y. (2005). Growth discrepancy in twins in the first trimester of pregnancy. *Ultrasound Obstet Gynecol, 26*(5), 512-516, doi:10.1002/uog.1966.

Sebire, N. J., D'Ercole, C., Hughes, K., Carvalho, M., & Nicolaides, K. H. (1997a). Increased nuchal translucency thickness at 10-14 weeks of gestation as a predictor of severe twin-to-twin transfusion syndrome. *Ultrasound Obstet Gynecol, 10*(2), 86-89, doi:10.1046/j.1469-0705.1997.10020086.x.

Sebire, N. J., D'Ercole, C., Soares, W., Nayar, R., & Nicolaides, K. H. (1998). Intertwin disparity in fetal size in monochorionic and dichorionic pregnancies. *Obstet Gynecol, 91*(1), 82-85.

Sebire, N. J., Snijders, R. J., Hughes, K., Sepulveda, W., & Nicolaides, K. H. (1996). Screening for trisomy 21 in twin pregnancies by maternal age and fetal nuchal translucency thickness at 10-14 weeks of gestation. *Br J Obstet Gynaecol, 103*(10), 999-1003.

Sebire, N. J., Snijders, R. J., Hughes, K., Sepulveda, W., & Nicolaides, K. H. (1997b). The hidden mortality of monochorionic twin pregnancies. *Br J Obstet Gynaecol, 104*(10), 1203-1207.

Senoo, M., Okamura, K., Murotsuki, J., Yaegashi, N., Uehara, S., & Yajima, A. (2000). Growth pattern of twins of different chorionicity evaluated by sonographic biometry. *Obstet Gynecol, 95*(5), 656-661.

Sepulveda, W., Wong, A. E., & Casasbuenas, A. (2009). Nuchal translucency and nasal bone in first-trimester ultrasound screening for aneuploidy in multiple pregnancies. *Ultrasound Obstet Gynecol, 33*(2), 152-156, doi:10.1002/uog.6222.

Shrim, A., Weisz, B., Gindes, L., & Gagnon, R. (2010). Parameters associated with outcome in third trimester monochorionic diamniotic twin pregnancies. *J Obstet Gynaecol Can, 32*(5), 429-434.

Singh, A., Singh, A., & Nirmalan, P. K. (2014). Associations for birthweight of twin pairs in south India. *J Obstet Gynaecol Res, 40*(1), 215-218, doi:10.1111/jog.12160.

Smith, A. P. M., Ong, S., Smith, N. C. S., & Campbell, D. (2001). A prospective longitudinal study of growth velocity in twin pregnancy. *Ultrasound in Obstetrics and Gynecology, 18*(5), 485-487, doi:<http://dx.doi.org/10.1046/j.0960-7692.2001.00519.x>.

Snijder, M. J., & Wladimiroff, J. W. (1998). Fetal biometry and outcome in monochorionic vs. dichorionic twin pregnancies; a retrospective cross-sectional matched-control study. *Ultrasound Med Biol, 24*(2), 197-201.

Sooranna, S. R., Ward, S., & Bajoria, R. (2001). Fetal leptin influences birth weight in twins with discordant growth. *Pediatr Res, 49*(5), 667-672, doi:10.1203/00006450-200105000-00010.

Spencer, J. V., Ingardia, C. J., Nold, C. J., Borgida, A. F., Herson, V. C., & Egan, J. F. (2009). Perinatal and neonatal outcomes of triplet gestations based on placental chorionicity. *Am J Perinatol, 26*(8), 587-590, doi:10.1055/s-0029-1220776.

Spencer, K. (2001). Screening for trisomy 21 in twin pregnancies in the first trimester: does chorionicity impact on maternal serum free beta-hCG or PAPP-A levels? *Prenat Diagn, 21*(9), 715-717.

Spencer, K., Kagan, K. O., & Nicolaides, K. H. (2008). Screening for trisomy 21 in twin pregnancies in the first trimester: an update of the impact of chorionicity on maternal serum markers. *Prenat Diagn, 28*(1), 49-52, doi:10.1002/pd.1923.

Steingass, K. J., Taylor, H. G., Wilson-Costello, D., Minich, N., & Hack, M. (2013). Discordance in neonatal risk factors and early childhood outcomes of very low birth weight (<1.5 kg) twins. *J Perinatol, 33*(5), 388-393, doi:10.1038/jp.2012.121.

Stiller, R. J., Lockwood, C. J., Belanger, K., Baumgarten, A., Hobbins, J. C., & Mahoney, M. J. (1988). Amniotic fluid alpha-fetoprotein concentrations in twin gestations: dependence on placental membrane anatomy. *Am J Obstet Gynecol, 158*(5), 1088-1092.

Suzuki, S., & Matsuhashi, T. (2007). Influence of chorionicity on umbilical arterial blood gas discordance in twin pairs following elective Cesarean delivery. *J Matern Fetal Neonatal Med, 20*(10), 773-775, doi:10.1080/14767050701510256.

Tagin, M. A., Vincer, M. J., Woolcott, C. G., & Allen, V. M. (2011). Long-term outcomes of singletons and twins <31 weeks at birth: A population-based study. [Conference Abstract]. *Paediatrics and Child Health, 16*, 9A.

Taylor, G. M., Owen, P., & Mires, G. J. (1998). Foetal growth velocities in twin pregnancies. *Twin Res, 1*(1), 9-14.

Tobe, R. G., Mori, R., Shinozuka, N., Kubo, T., & Itabashi, K. (2010). Birthweight discordance, risk factors and its impact on perinatal mortality among Japanese twins: data from a national project during 2001-2005. *Twin Res Hum Genet, 13*(5), 490-494, doi:10.1375/twin.13.5.490.

van Steenis, A., Kromhout, H. E., Steggerda, S. J., Sueters, M., Rijken, M., Oepkes, D., et al. (2014). Perinatal asphyxia in monochorionic versus dichorionic twins: incidence, risk factors and outcome. *Fetal Diagn Ther, 35*(2), 87-91, doi:10.1159/000356433.

Vergani, P., Locatelli, A., Ratti, M., Scian, A., Pozzi, E., Pezzullo, J. C., et al. (2004). Preterm twins: What threshold of birth weight discordance heralds major adverse neonatal outcome? [Conference Paper]. *Am J Obstet Gynecol, 191*(4), 1441-1445, doi:<http://dx.doi.org/10.1016/j.ajog.2004.05.053>.

Vergani, P., Russo, F., Follesa, I., Cozzolino, S., Fedeli, T., Ventura, L., et al. (2013). Perinatal complications in twin pregnancies after 34 weeks: Effects of gestational age at delivery and chorionicity. *Am J Perinatol, 30*(7), 545-550, doi:<http://dx.doi.org/10.1055/s-0032-1329183>.

Victoria, A., Mora, G., & Arias, F. (2001). Perinatal outcome, placental pathology, and severity of discordance in monochorionic and dichorionic twins. *Obstet Gynecol, 97*(2), 310-315.

Visentin, S., Grisan, E., Zanardo, V., Bertin, M., Veronese, E., Cavallin, F., et al. (2013). Developmental programming of cardiovascular risk in intrauterine growth-restricted twin fetuses according to aortic intima thickness. *J Ultrasound Med, 32*(2), 279-284.

Welch, P., Black, K. N., & Christian, J. C. (1978). Placental type and Bayley Mental Development scores in 18-month-old twins. *Prog Clin Biol Res, 24a*, 145-149.

Westwood, M., Gibson, J. M., Sooranna, S. R., Ward, S., Neilson, J. P., & Bajoria, R. (2001). Genes or placenta as modulator of fetal growth: Evidence from the insulin-like growth factor axis in twins with discordant growth. *Mol Hum Reprod, 7*(4), 387-395.

Yu, C. K., Papageorghiou, A. T., Boli, A., Cacho, A. M., & Nicolaides, K. H. (2002). Screening for pre-eclampsia and fetal growth restriction in twin pregnancies at 23 weeks of gestation by transvaginal uterine artery Doppler. *Ultrasound Obstet Gynecol, 20*(6), 535-540, doi:10.1046/j.1469-0705.2002.00865.x.
